# Cryo-EM structures and transport mechanism of human P5B type ATPase ATP13A2

**DOI:** 10.1038/s41421-021-00334-6

**Published:** 2021-11-02

**Authors:** Xudong Chen, Mingze Zhou, Sensen Zhang, Jian Yin, Ping Zhang, Xujun Xuan, Peiyi Wang, Zhiqiang Liu, Boda Zhou, Maojun Yang

**Affiliations:** 1 Ministry of Education Key Laboratory of Protein Science, Tsinghua-Peking Center for Life Sciences, Beijing Advanced Innovation Center for Structural Biology, School of Life Sciences, Tsinghua University, Beijing, China; 2 Department of Cardiology, Beijing Tsinghua Changgung Hospital, School of Clinical Medicine, Tsinghua University, Beijing, China; 3 Department of Andrology, The Seventh Affiliated Hospital, Sun Yat-sen University, ShenZhen, Guangdong China; 4 Cryo-EM Facility Center, Southern University of Science & Technology, Shenzhen, Guangdong, China; 5 Department of Anesthesiology, Shanghai First Maternity and Infant Hospital, School of Medicine, Tongji University, Shanghai, China

**Keywords:** Cryoelectron microscopy, Transport carrier

## Abstract

Polyamines are important polycations that play critical roles in mammalian cells. ATP13A2 belongs to the orphan P5B adenosine triphosphatases (ATPase) family and has been established as a lysosomal polyamine exporter to maintain the normal function of lysosomes and mitochondria. Previous studies have reported that several human neurodegenerative disorders are related to mutations in the ATP13A2 gene. However, the transport mechanism of ATP13A2 in the lysosome remains unclear. Here, we report the cryo-electron microscopy (cryo-EM) structures of three distinct intermediates of the human ATP13A2, revealing key insights into the spermine (SPM) transport cycle in the lysosome. The transmembrane domain serves as a substrate binding site and the C-terminal domain is essential for protein stability and may play a regulatory role. These findings advance our understanding of the polyamine transport mechanism, the lipid-associated regulation, and the disease-associated mutants of ATP13A2.

## Introduction

Parkinson’s disease (PD) is the second most common progressive neurodegenerative disease characterized by progressive disturbances in motor, autonomic, and psychiatric functions^1,2^. To date, around ten genes have been identified to contribute to the hereditary PD, including the S*NCA, PRKN, UCH-L1, PINK1, DJ-1, LRRK2, ATP13A2, HtrA2, PLA2G6*, and *FBX07*^3,4^. Kufor-Rakeb syndrome (KRS) is an early-onset autosomal recessive form of PD with dementia, spasticity, and cognitive decline^5,6^, which is implicated by the mutation of ATP13A2 (PARK9)^7,8^. Moreover, *ATP13A2* mutations were also reported in neuronal ceroid lipofuscinosis (NCL)^9^, amyotrophic lateral sclerosis (ALS)^10^, and hereditary spastic paraplegia (HSP)^11^. Knockdown of ATP13A2 leads to lysosomal dysfunctions accompanied by impaired autophagosomal flux, and accumulation of fragmented mitochondria, whereas overexpression of ATP13A2 suppresses α-synuclein toxicity, highlighting the central role of ATP13A2 in PD^12–16^.

ATP13A2 belongs to the P5B ATPases and functions as a late endolysosomal transporter that is widely expressed in various tissues, especially with the highest expression in the brain^7^. P-type ATPases constitute a large protein family that pumps substrates across cellular membranes through conformational change coupled with ATP hydrolysis, which can be further classified into five distinct subfamilies (P1–P5)^17,18^. The P1- to P3-ATPases are well-characterized ion pumps, whereas the P4-ATPases function as lipid flippase to mediate the translocation of phospholipids^19,20^.

The P5-ATPases could be divided into two subgroups (P5A and P5B) based on phylogenetic analysis and different intracellular localization^21,22^. Recently, Spf1, the single P5A-ATPase in yeast, was identified as a transmembrane helix dislocase^23^. For a long time, little is known concerning the P5B-ATPase subgroup but genetic lesions in some members cause neurological diseases, including the most well-characterized member ATP13A2 in humans. Initially, ATP13A2 was suggested to function as a cation transporter to translocate the heavy metal ions^24^. However, later studies proposed that ATP13A2 mediates spermidine (SPD) uptake and is crucial for norspermidine-mediated suppression of RNA interference^25,26^. Recently, ATP13A2 was established as a polyamine exporter with a higher affinity for SPM than SPD, thus protecting the cell from being damaged by high concentration polyamines toxicity^27^. In addition, the structures of Ypk9, a yeast homolog of mammalian ATP13A2-5, were reported to provide insights into the yeast polyamine transport mechanism^28^.

Although the properties of lipid interaction, substrate translocation, and disease-related mutations have been investigated, the molecular mechanism and Parkinsonism pathogenesis have remained elusive due to the lack of structural information. Here, using single-particle cryo-EM, we present the structures of full-length human ATP13A2 in three distinct conformations including an E1 state (apo), an E1P-ADP state with ATP and Mg^2+^, and an E2-Pi state with SPM, phosphatidic acid (PA), and phosphatidylinositol(3,5)bisphosphate [PI(3,5)P2]. These structures not only reveal the key insights into the mechanism of SPM transport in the lysosome membrane but also expand our understanding of the function of the P5B ATPase subfamily.

### Structure determination and overall structure of ATP13A2

**Fig. 1:**
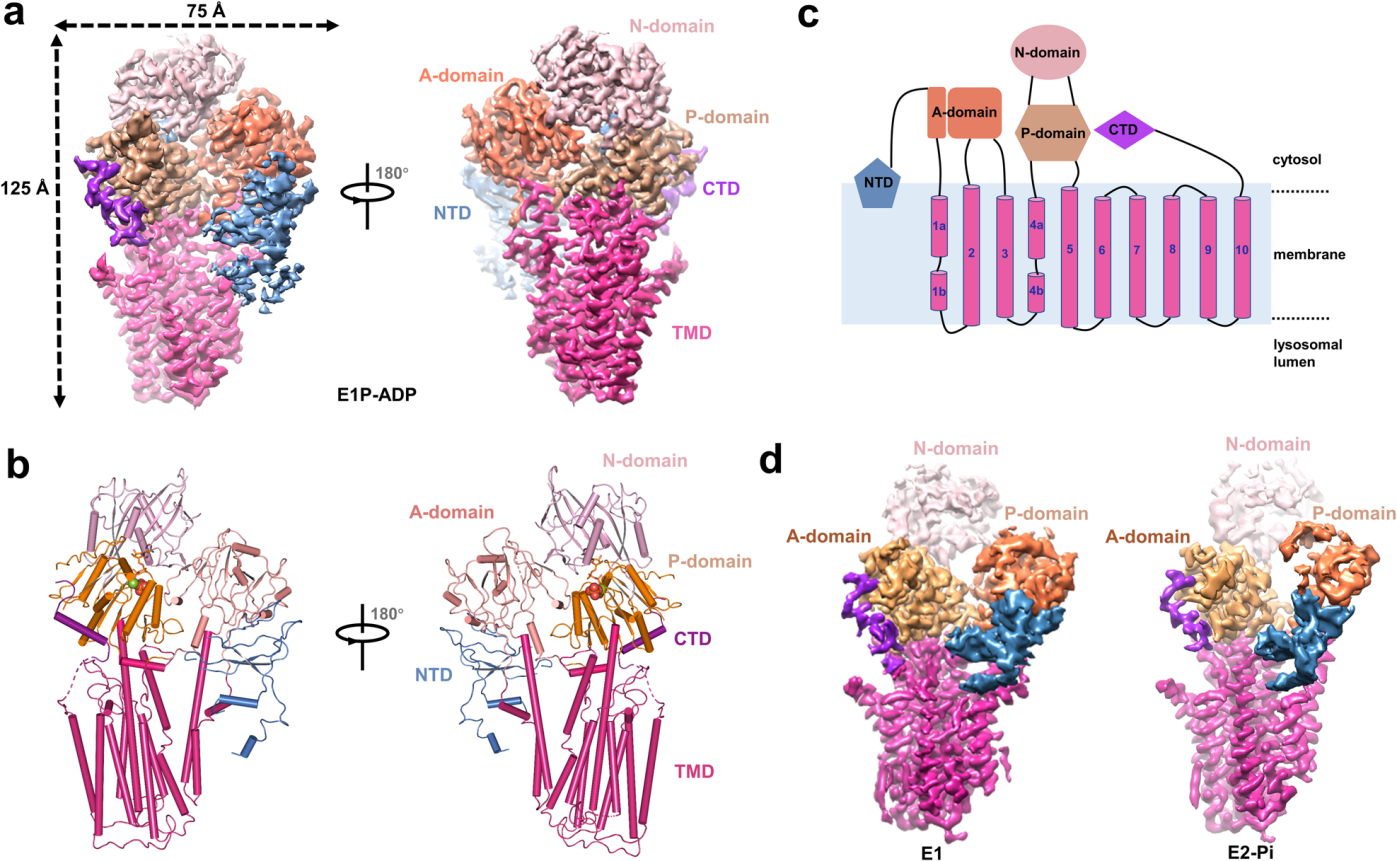
Overall structure of human ATP13A2. **a**, **b** 3.0-Å-resolution cryo-EM map (**a**) and ribbon model (**b**) of human ATP13A2 in E1P-ADP state. The A, N, and P domains and the transmembrane domain are colored orange, rose, brown, and magenta, respectively. The N-terminus domain and C-terminus extension domain of ATP13A2 are blue and purple. The same color scheme is used throughout this manuscript. The ADP and Pi are shown as spheres. **c** Topology diagram of human ATP13A2. Conserved domains and TM helices are schematically illustrated. Note that the helix of TM4 is unraveled halfway by a P5B-ATPase conserved PP(A/V)LP motif. **d** Cryo-EM maps of human ATP13A2 in E1 state and E2-Pi state.

To obtain homogeneous proteins suitable for cryo-EM study, human ATP13A2 was overexpressed in mammalian cells using a strep affinity tag and subsequently reconstituted in digitonin micelle buffer (Supplementary Fig. S1). The purified human ATP13A2 was subjected to cryo-EM analysis under three different conditions, apo state, E1P-ADP state (with Mg^2+^, ATP, and SPM), and E2-Pi state [with PI(3,5)P2, PA, and SPM], and at overall resolutions of 3.3, 3.0, and 3.6 Å, respectively (Fig. 1, Supplementary Figs. S2–S4 and Table S1). The E1P-ADP state map showed well-resolved densities to allow confident *de novo* model building for most of the protein regions (Supplementary Fig. S5), the other two state models were reconstructed according to the E1P-ADP state structure.

The structure of the human ATP13A2 adopts the typical P-type ATPase fold with overall dimensions of 125 Å × 75 Å × 70 Å, including ten transmembrane (TM) helices and three conserved cytoplasmic domains [N (nucleotide-binding) domain; A (actuator) domain; and P (phosphorylation) domain]. In addition, the P5B type ATP13A2 also comprises two specific domains: a C-terminal extension domain (CTD) laying close to the P-domain and an N-terminal domain (NTD) preceding the A domain showing amphipathic feature (Fig. 1). To describe the overall architecture and the specific domains, we will reference the 3.0-Å-resolution E1P-ADP state structure because the flexible cytosolic domains are better resolved in this map.

### CTD of ATP13A2

**Fig. 2:**
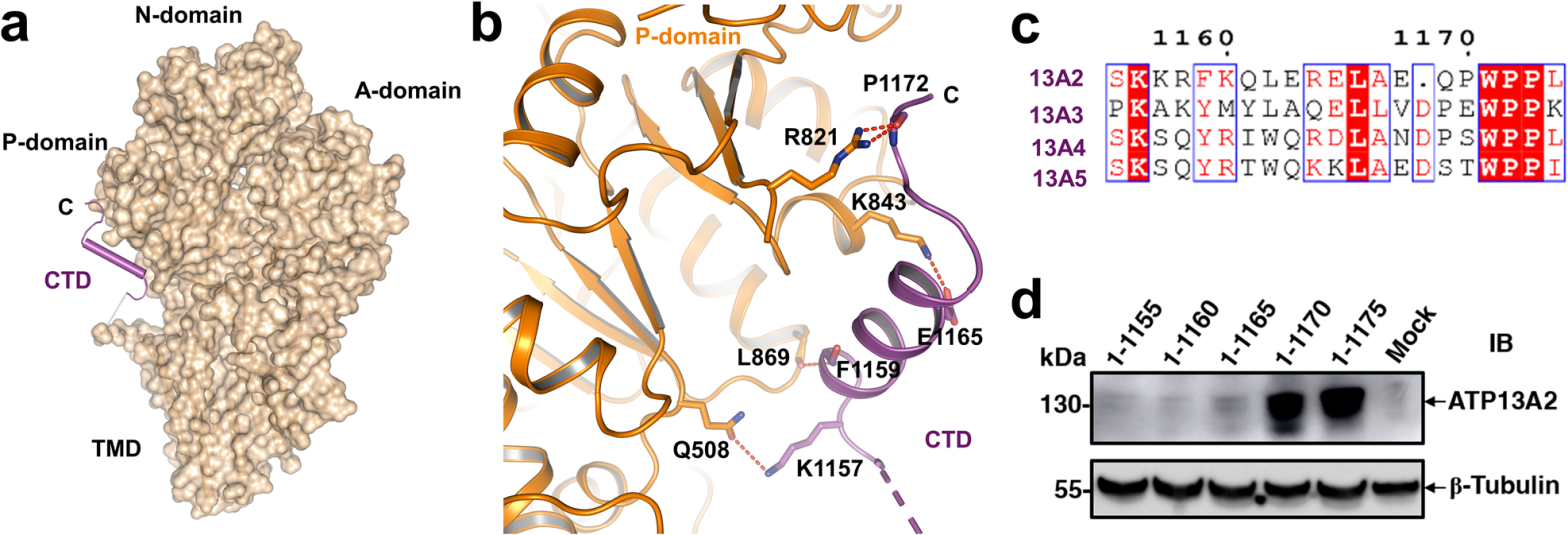
CTD of ATP13A2 interacts with P-domain, affects protein expression and stability. **a** Overall structure of E1P-ADP state ATP13A2 in surface representation. The CTD is shown as a purple stick model. **b** Ribbon representation of the domain interface between CTD and P-domain. Polar interactions are shown as red dashed lines. **c** Sequence alignment of the CTD of human ATP13A2 to ATP13A5 using ESPript3. Residues are considered as highly similar are colored in red and framed in blue. **d** Immunoblotting of HEK293F overexpressing five ATP13A2 CTD deletion truncations (5, 10, 15, 20, and 25 amino acids were deleted from C-terminus, respectively).

A previous study^29^ has identified a nonsense mutation (Q1135*), through which ATP13A2 loses the autophosphorylation activity, accompanied with complicated HSP. Notably, this mutation occurs at the CTD of ATP13A2. It has been suggested that many P-type ATPases utilize the terminal ends to act as a regulatory domain to inhibit the domain movements, to sense the transported cations, or to regulate the pump cation affinities^17^. In our E1P-ADP state structure, different from ATP13A1^23^, the CTD of ATP13A2 consists of a helix and link loops, extending from the TM10 to the cytosol and laying close to the P domain. The C terminal helix interacts with the neighboring P domain via several interactions, including the hydrogen-bonds interactions via P1172-R821, E1165-K843, F1159-L869, and K1157-Q508 (Fig. 2a, b). Sequence alignment of CTD indicates that many interacting residues are not well conserved among ATP13A2 to A5 (Fig. 2c). The CTD heterogeneity among P5B subfamily may be related to the organ-specific functions of these proteins since the expression levels of these P5B ATPases vary considerably between different tissues^30,31^ and the currently known human diseases associated with each of them are different^32–35^.

To investigate the potential role of the CTD, we performed sequential truncation by five consecutive residues from the C terminal of ATP13A2. The protein expression levels of the resultant mutants, including 1-1155, 1-1160, and 1-1165 truncations, were largely impaired, whereas the 1-1170 and 1-1175 truncations retain the normal expression level (Fig. 2d). Our results are consistent with the reported nonsense mutation (Q1135*; equivalent to Q1140* in the current hATP13A2 model) since a similar impaired expression level was observed in Q1135* mutant^29^, indicating that CTD may play an important role in maintaining protein stability in vivo. It is intriguing that the reported nonsense mutation has impaired the ability of autophosphorylation of ATP13A2, raising the possibility that CTD is related to the phosphorylation regulation of this ATPase by interacting with the P domain.

### The E1 and E1P-ADP state of ATP13A2

P-type ATPase undergoes autophosphorylation to couple ATP hydrolysis to transport substrate^17^. A previous study indicates that ATP13A2 undergoes autophosphorylation and accumulates in the E1P state^36^. To investigate the conformational change of ATP13A2 during the phosphorylation process, we solved two intermediate state structures, a 3.3 Å resolution E1 state and a 3.0 Å E1P-ADP state. Both of these two maps show well-resolved density.

**Fig. 3:**
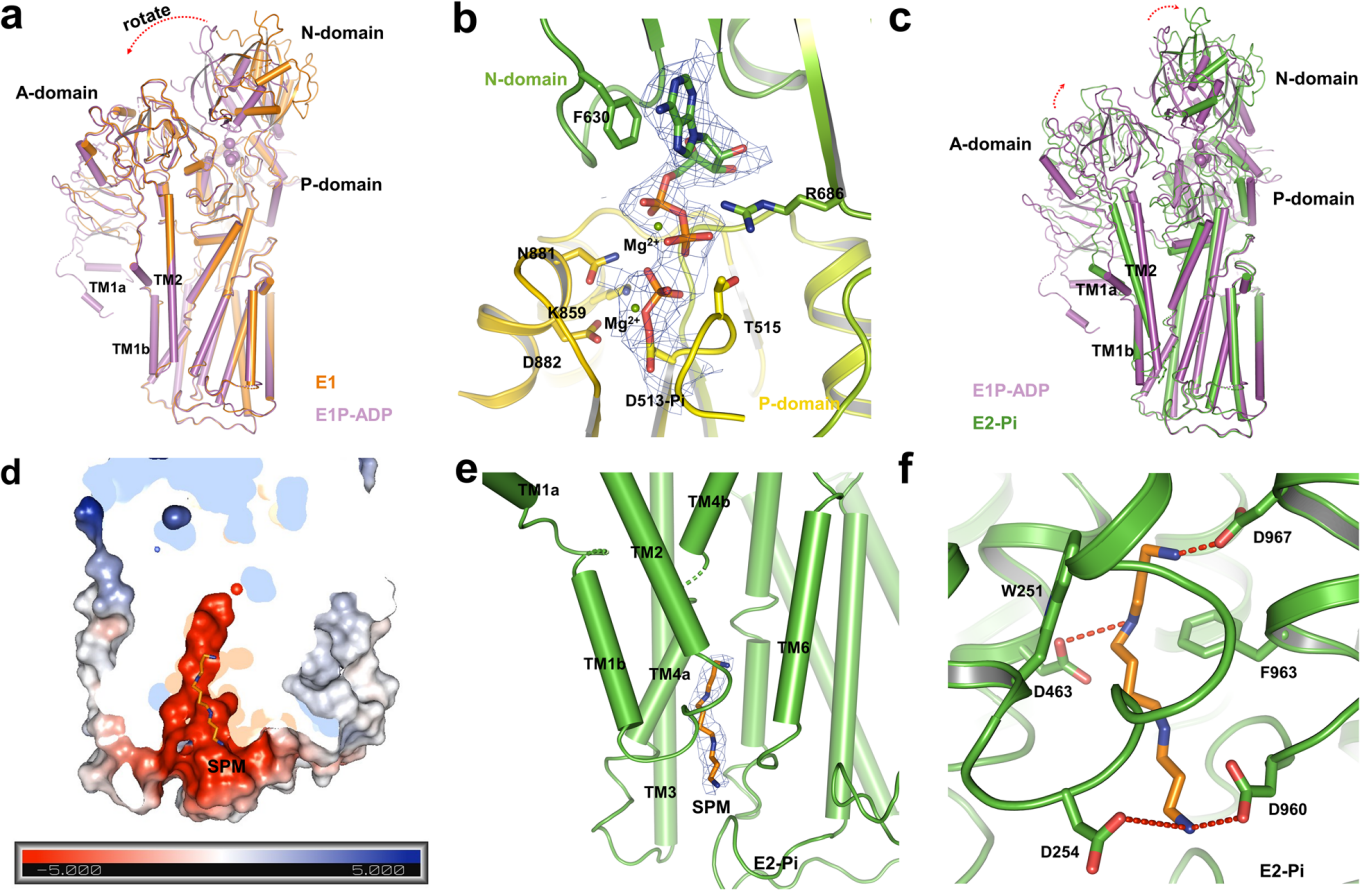
Comparisons of different intermediate states and substrate-binding site. **a** Conformational changes of ATP13A2 from the E1 state (orange) to the E1P-ADP state (purple) are shown. **b** Close-up view of the phosphorylation sites in E1P-ADP state. ADP and Pi are shown as sticks, and Mg^2+^ are shown as spheres. Densities are shown as blue mesh, contoured at 5 *σ*. **c** Conformational changes of ATP13A2 from the E1P-ADP state (purple) to the E2-Pi state (green) are shown. **d** Heatmap of the surface electrostatics of TMs shows a substrate-binding cavity. The SPM molecule is shown as sticks. **e** Cryo-EM density showing the bound substrate SPM (blue mesh, 6 *σ*). The SPM molecule is shown as sticks. **f** Ribbon representation of the interaction of TMs with SPM. The SPM molecule is shown as sticks.

ATP binds to ATP13A2 and the resultant phosphoryl transfer reaction induces the rearrangement of the N domain, by which the N domain tilt around 30° to lay close to the A and P domain, thus sealing the crevice formed by the N and P domain compared to the E1 state (Fig. 3a). After the E1-ATP-to-E1P-ADP transition, ADP molecular was stabilized by several polar interactions, in which the adenine ring of the ADP forms interaction with F630 from the N domain via π–π interaction, whereas the phosphate group of ADP interacts with the neighboring N881, K859, D882, and T515 from the P domain via hydrogen-bonds interactions. In addition, two Mg^2+^ ions were coordinated by the carbonyl of T515 and phosphorylated D513 (Fig. 3b). Mutagenesis of the aspartate (D513A) resulted in a reduced activity compared with wild-type proteins, highlighting the essential role of this conserved aspartate in ATP13A2 activity (Supplementary Fig. S6).

### The substrate binding E2-Pi state of ATP13A2

A previous study identified that SPM functions as a substrate of ATP13A2^27^. To investigate the possible SPM translocation mechanism of ATP13A2, we added SPM, PA, and PI(3,5)P2 during protein purification and sample preparation, and ultimately, we solve the structure of the E2-Pi state at a resolution of 3.6 Å. Comparison between this state and the E1P-ADP state reveals a door sliding motion formed by TM1 and TM2 accompanied by the rotation of the A domain and the tilt of the N domain (Fig. 3c). The conformational change in TMD creates a spindly negatively charged cavity that allows substrate entry from the lysosomal matrix (Fig. 3d). Notably, in our E1 state and E1P-ADP state structures, this cavity is nearly closed which prevents the binding and passage of substrate (Supplementary Fig. S7). This may explain why we cannot find the SPM density in our E1P-ADP map though we have already added SPM in cryo-sample preparation. In this cavity forms by TM1b, TM2, TM4a, and TM6, we observed an extra density that extends from the lysosomal lumen to the TMD. This density was assigned as an SPM molecule (Fig. 3d, e). The SPM molecule is stabilized by extensive interactions between the amine nitrogen atoms groups from SPM and the acidic and aromatic side chains along the cavity. For instance, the amine nitrogen atoms are coordinated by D254, D463, D960, and D967 via hydrogen bonding interactions and by W251 and F963 via cation–π interactions (Fig. 3f).

### Comparisons between different P5-ATPases

**Fig. 4:**
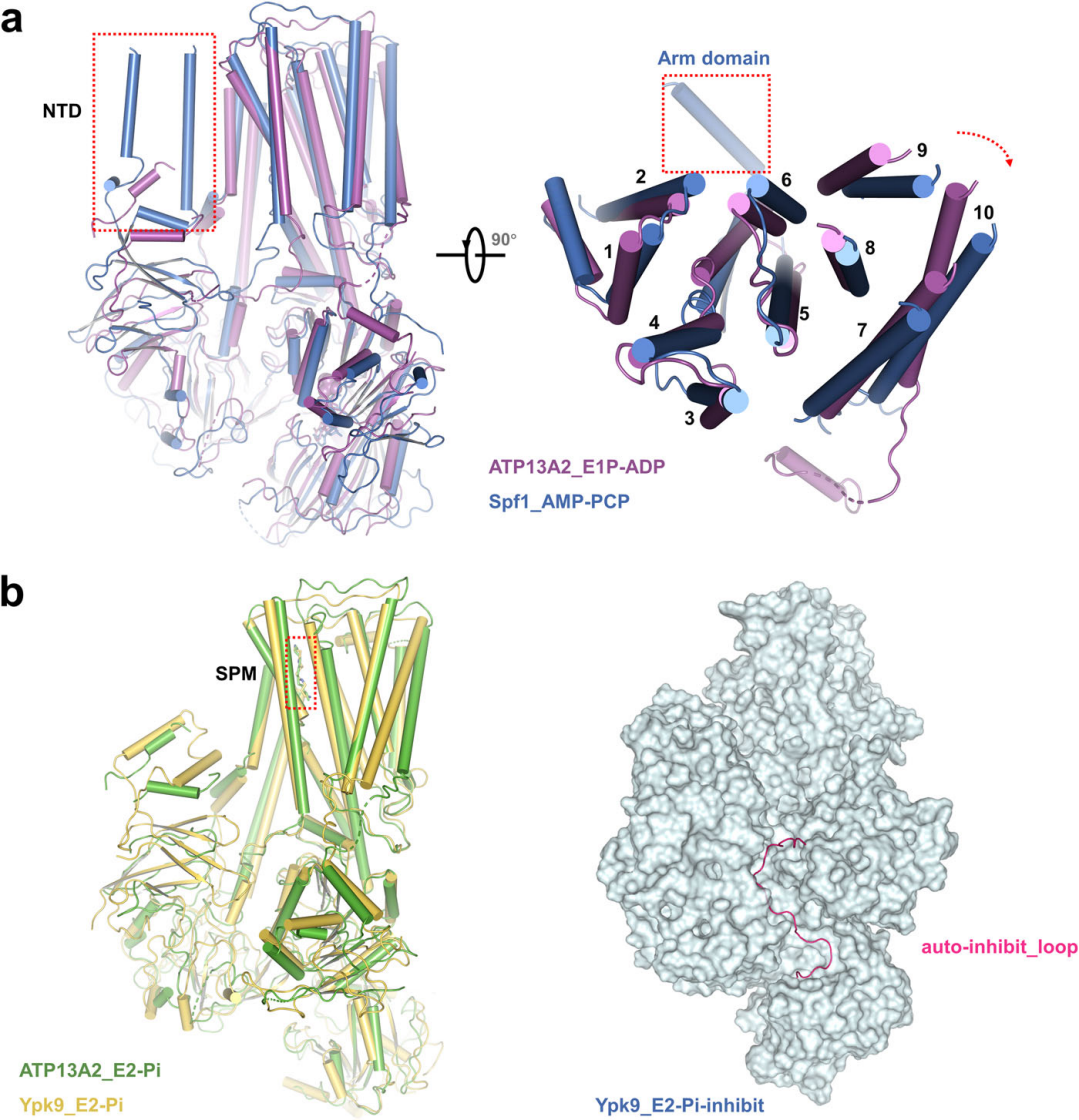
Structural comparisons of ATP13A2 with yeast P5A ATPase Spf1 and P5B ATPase Ypk9. **a** Structural comparison of human ATP13A2 in E1P-ADP state (purple) and Spf1 in E1-AMPPCP state (blue). The N-terminal Arm domain of Spf1 is shown in the red dashed box. **b** Structural comparison of human ATP13A2 in E2-Pi state (green) and Ypk9 in E2-Pi state (yellow) (left). Surface representation of the Ypk9 in E2-Pi-inhibit state, the auto-inhibit loop is colored magenta (right).

A previous study^23^ has revealed the pivotal role of P5A-ATPase (Spf1, a yeast homolog of human ATP13A1) in ensuring correct mitochondrial tail-anchored protein localization, which strands contrast to the polyamine transport function of human ATP13A2 at lysosomes. Structure comparisons between the P5A-ATPase Spf1 and the P5B-ATPase human ATP13A2 indicate that these two transporters share similar structure elements except that human ATP13A2 lacks the arm domain and N terminal transmembrane helix in Spf1 (Fig. 4a). Previous studies uncovered the essential role of the hydrophobic N-terminal, through which ATP13A2 interacts with the signaling lipids PA and PI(3,5)P2, thus unlocking the activity of ATP13A2^36,37^. The N-terminal cryo-EM densities of these P5B-ATPase structures are weaker compared with those of Spf1 structures (Supplementary Figs. S2-S4), largely due to interaction with endogenous signaling lipids, albeit it is hard to assign the location of these molecules in our current structures. In addition, human ATP13A2 contains a narrow tunnel ― formed by TMs 1, 2, 4, 5, 6 ― than that of Spf1, due to the spindly character of the spermine substrate (Fig. 4a). The clockwise rotation of the other helices in Spf1, including TMs 7, 8, 9, and 10, promotes the open of the binding pocket for accommodating a much bigger substrate, like a mistargeting polypeptide (Fig. 4a).

Among the same P5B subtype ATPases, human ATP13A2 shares a similar secondary structure fold to the yeast homolog Ypk9, with an RMSD of 1.8 Å over 674 Cα atoms (Fig. 4b). The binding manner of the SPM substrate in these two structures is highly conserved. Interestingly, the E2P-inhibit state of Ypk9 reveals a long N terminal loop that interacts with the A- and N-domains and hijacks the ATPase in the E2P state. Such an auto-inhibition regulation mechanism is not observed in our current human ATP13A2 structures. Sequence alignment of human ATP13A2 and Ypk9 also corroborate this since human ATP13A2 lacks this homologous N terminal loop (Supplementary Fig. S8). This regulation discrepancy between different species enriched our understanding of P5B ATPase regulation during the evolution process.

### Structure interpretation of the pathogenesis mutations

Recent evidence has tied around 40 ATP13A2 mutations to many neurodegenerative disorders, including the PD^8^, KRS^7^, HSP^11^, NCL^9^, and ALS^10^, for which the etiology and pathogenesis remain elusive (Supplementary Table S2). Our current structures of ATP13A2 provide a framework to mechanistically understand the pathogenesis of these life-threatening diseases. Among them, 30 disease-related mutations could be mapped into the current structure (Supplementary Fig. S9a), which could be further divided into three catalogs by correlating these mutations with their functional consequences.

Mutations located in the TMD regions were expected to affect the substrate translocation. For instance, three single point mutations, A249V, R449Q, and R980H which were identified in PD patients^38^, locate very close to the substrate binding and transport cavity in our ATP13A2 structure and are likely to affect substrate binding and the following translocation from the lysosomal lumen to the cytoplasm, leading to the over-accumulation of spermine in the lysosome, which is an important predisposing factor for PD (Supplementary Fig. S9b). Among the NTD and A domain, W71X and Q122* were expected to disrupt the interaction with the PI(3,5)P2 molecules, whereas the F182L might affect the binding of the PA molecules during ATPase translocation (Supplementary Fig. S9c). In addition, a cluster of residue mutations (T517I, F851Cfs, L825fs, A855D, G877R, etc.) were reported near the interface between the P and N domain, through which the mutants will affect the autophosphorylation of the ATP13A2 (Supplementary Fig. S9d). In addition, ATP13A2 was also correlated with the maintenance of mitochondrial function and morphology, since two mutations were observed in ATP13A2 accompanied with increased mtDNA levels, more fragmented mitochondrial network, and respiratory chain dysfunction^13^. Moreover, a recent study identifies a protective pathway that counters mitochondrial oxidative stress via ATP13A2-mediated lysosomal spermine export^39^. It is anticipated that further understanding of the ATP13A2 mechanism will help delineate the relationship between ATP13A2 and mitochondria function.

### Proposed mechanism of polyamine transport by ATP13A2 in lysosome

Polyamines are a class of important polycations that play essential roles in protein synthesis, protection from oxidative damage, activity of ion channels, cell proliferation, differentiation, and apoptosis in mammalian cells and are tightly regulated by complex processes including synthesis, degradation, and transport^40^. Identified as a polyamine transporter in lysosome, ATP13A2 plays important role in maintaining the polyamine homeostasis through its transport activity^27^. In this study, we resolved structures of three distinct intermediated states in the ATP13A2 transport cycle and uncovered the substrate-binding site of ATP13A2. These results together reveal a proposed model for SPM transport by ATP13A2 in lysosome.

**Fig. 5:**
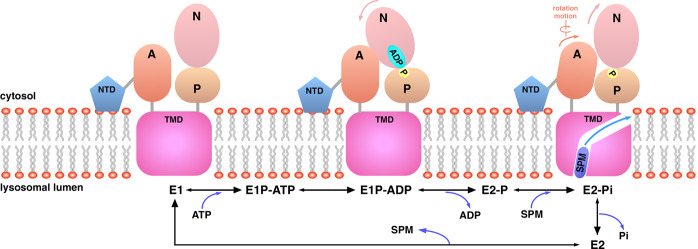
Proposed mechanism of SPM transport by ATP13A2. Schematic model of the SPM transport cycle by ATP13A2, according to the Post-Albers mechanism. The model is depicted with the same colors as in Fig. 1a and colored arrows indicate the coming movements of the corresponding domain to the following conformation.

The binding of ATP and Mg^2+^ induces the proximal arrangement of the N domain, by which the N domain tilt around 30° to lay close to the A and P domain, resulting in the E1-to-(E1-ATP) transition. After the phosphoryl transfer reaction, ADP is released from the N domain, leading to the E1P-ADP-to-E2P transition. In the E2P state, a substrate binding cavity formed by TMs is generated. The E2P to E2-Pi transition is accompanied by the rearrangement of the A domain and the binding of SPM molecule in lysosomal lumen to the substrate cavity in the TMD of ATP13A2, thereby facilitating the dephosphorylation reaction. Release of the Pi forms the intermediate E2 state, in which the cavity towards cytosol is open, thus allowing the translocation of SPM to the cytosol side. The A-domain should move back to the original position after the release of SPM to the cytosol, the protein then adopts the E1 conformation, ready to initiate another transport cycle (Fig. 5).

## Discussion

A previous study reported that PA and PI(3,5)P2 interact with the NTD truncation (1-187) of ATP13A2, revealing important roles of the two regulatory lipids in unlocking the activity of ATP13A2^37^. In our ATP13A2 structure, we observed a small positively charged area in the NTD comprising the residues K160 and R161 (Supplementary Fig. S10a). This positively charged area was proposed to play role in recruiting the negatively charged regulatory lipids^37^. To verify our speculation, we mutated the positively charged residues and performed an ATPase assay using purified human full-length ATP13A2 proteins (wild-type or mutant). The addition of PA molecules leads to increased activation of ATP13A2, highlighting the essential role of PA in the activation of ATP13A2. Mutagenesis of the positively charged residues in the double-residues mutant (K160A/R161A) abolishes the PA-dependent activation manner of ATP13A2, albeit the mutant does not affect protein expression (Supplementary Fig. S10b, c). Our results feature the potential binding site of the regulatory PA in ATP13A2.

In addition, in order to check whether ATP13A2 also interacts with other lipids, human full-length ATP13A2 was used to screen in vitro for lipid interactions utilizing a protein-lipid overlay assay containing a total of 22 lipids. Interestingly, except PA and PI(3,5)P2, we also found other lipids show more or less binding affinity with ATP13A2, such as PI(3)P, PI(4)P, PI(5)P, and PI(4,5)P2 (Supplementary Fig. S10d, e). These negatively charged phosphatidylinositols might be recruited by the strongly positively charged surface not only in the NTD but also in other domains, functioning in the regulation of ATP13A2 activity. Given the complexity of lipids-associated activation of ATP13A2, more studies are needed in the future.

The P5B ATPase diversified from one isoform in fungi and primitive animals to a maximum of four in mammals by successive gene duplication events in vertebrate evolution^41^. Comparison of human ATP13A2-ATP13A5 reveals 36% (A2 and A5) to 57% (A4 and A5) amino acid identity (Supplementary Fig. S8). ATP13A2 and ATP13A3 presumably duplicated from a common P5B ancestor gene, and the long evolutionary separation between them resulted in significant sequence diversity and two clearly separated clades^41^. In humans, ATP13A2 and ATP13A3 share 39% amino acid identity and they are widely expressed in various tissues, with ATP13A2 predominantly expressed in the brain and ATP13A3 mainly expressed in the liver^7,30^. Recently, ATP13A3 was supposed to contribute to putrescine uptake in a mammalian cell line, indicating that ATP13A3 may also function as a polyamine transporter^42^. Since ATP13A3 was also found to involve in human diseases such as heritable pulmonary arterial hypertension (HPAH)^32,33,43^, both ATP13A2 and ATP13A3 may act as important components of the mammalian polyamine transport system and were implicated in various human diseases.

For the other two P5B ATPase, ATP13A4 and ATP13A5, they not only share the highest 57% amino acid identity but also display a more limited tissue distribution in the brain and epithelial glandular cells^30,41^. In particular, ATP13A4 was found to localize to the endoplasmic reticulum^35^ and implicate in a range of psychiatric disorders, such as autism spectrum disorders (ASD)^44,45^ and specific language impairment (SLI)^34^. ATP13A4 and ATP13A5 may also act as members of the polyamine transport system, but display a more specific cell and tissue expression. During the course of the preparation of this manuscript, the structures of human ATP13A2 in series of states using different protein isoform (isoform B), protein expression system (Sf9 cells), and protein purification method (in DDM/CHS micelles) have been reported as preprint^46^, revealing a similar polyamine transport mechanism to ours. Future studies will address the substrate specificity and structural information of other P5B ATPases, to demonstrate their function and mechanism in human polyamine transport.

For a long time, various metal ions were considered as transported substrates for the ATP13A2 because much solid evidence shows that APT13A2 is not only involved in the homeostasis of these metal ions but also provides protection against heavy metal toxicity^14–16,47–49^. However, recent studies indicated that these metal ions exert no effect on ATP13A2 activity^27,37^. In addition, our biochemical experiments show a very high ATPase activity when using SPM as substrate, and our structural results reveal the binding site of the SPM and the putative mechanism of SPM transport in lysosome. This evidence together certified ATP13A2 as a polyamine transporter. The function of ATP13A2 in stabilizing ion homeostasis and protecting heavy metal toxicity may associate with the polyamine transport process since polyamines play important roles in heavy metal chelating^50^ and act as antioxidant^51^ in cells.

In conclusion, our findings reveal the molecular mechanism of SPM transport by ATP13A2, which not only expands our understanding of the function of the P5B ATPase subfamily but also provides new insights into the human polyamine transport system. In addition, our findings on the activity regulation of ATP13A2 through lipids binding to NTD may offer a modality to therapeutically activate the ATP13A2 function to protect against neurodegenerative diseases.

## Materials and methods

### Materials

The following reagents were purchased from Sigma-Aldrich: NaCl (S9888), MgCl_2_ (M1028), HEPES [4-(2-Hydroxyethyl)piperazine-1-ethanesulfonic acid; H3375], spermine (S3256), ATP (adenosine 5'-triphosphate disodium salt hydrate; A2383), DTT (1,4-dithiothreitol; D0632), EDTA (ethylenediaminetetraacetic acid; E9884)

In addition, protease Inhibitor Cocktail Tablets (04693116001) were obtained from Roche. Digitonin (D3203) was obtained from BIOSYNTH. Strep-Tactin resin (2-1208-500) and D-desthiobiotin (2-1000-005) were ordered from IBA. 18:1 phosphatidic acid [1,2-dioleoyl-sn-glycero-3-phosphate (sodium salt); 840875] and 18:1 PI(3,5)P2 [1,2-dioleoyl-sn-glycero-3-phospho-1'-myo-inositol-3',5'-bisphosphate (ammonium salt); 850154] were purchased from Avanti Polar Lipids. For cell culture, Human embryonic kidney (HEK) 293 F cells (R79007) were purchased from Thermo Fisher Scientific Inc., the cell culture medium (SMM 293-TII) was obtained from Sino Biological Inc., and the Penicillin–Streptomycin Solution (SV30010) were ordered from Hyclone. We obtained the Polyethylenimine (PEI, Linear, MW 25000; 23966) from Polysciences.

### Cell culture and transfection

The optimized coding cDNA for Homo sapiens ATP13A2 isoform A (Uniprot: Q9NQ11-1) including a C-terminus tandem twin Strep-tag was cloned into the pcDNA3.1(-) vector for mammalian expression. HEK293F cells were cultured in medium supplemented with 1 × penicillin/streptomycin in a Multitron-Pro shaker (Infors, 120 r.p.m.) at 37 °C with 5% CO_2_.

To produce intact ATP13A2 proteins, 1 mg plasmids was pre-incubated with 2.5 mg PEI in 50 mL fresh medium for 25 m prior to adding the mixture to 1-L cells when cell density reached 1.5 × 10^6^ per milliliter. The transfected cells were cultured for 60 h before harvesting.

### Protein expression and purification for cryo-EM analysis

In order to obtain ATP13A2 protein in different conformational states, several batches of protein purifications were performed. For one batch of protein purification, about 5-L of transfected cells were harvested by centrifugation at 3,000 × g. All procedures below are carried out at 4 °C or on ice. Harvested cells were resuspended in lysis buffer containing 25 mM HEPES (pH 7.4), 150 mM NaCl, 2 mM DTT, and protease inhibitor, and then lysed by a high-pressure homogenizer. After removal of cell debris by centrifugation at 10,000 × g for 45 m, cell membrane fraction was pelleted by a 150,000 × g ultracentrifugation for 1 h. The membrane fraction was resuspended and solubilized in lysis buffer plus 1% (w/v) digitonin for 2 h with gentle rotation. After ultracentrifugation at 150,000 × g for 30 m, the supernatant was passed through a column filled with Strep-Tactin Sepharose resin. The resin was washed 50 column volume (CV) with wash buffer containing 25 mM HEPES (pH 7.4), 150 mM NaCl, 2 mM DTT and 0.1% (w/v) digitonin. The target ATP13A2 protein was eluted with wash buffer plus 10 mM desthiobiotin. The eluted ATP13A2 protein was concentrated to a final volume of ~ 100 μl by a 100 kDa cut-off centrifugal filter (Millipore) and further purified by size-exclusion chromatography (SEC) (Superose 6 5/150, GE Healthcare) in SEC buffer containing 25 mM HEPES (pH 7.4), 150 mM NaCl and 0.1% (w/v) digitonin. The SEC fractions corresponding to ATP13A2 were collected and verified by sodium dodecyl sulfate-polyacrylamide gel electrophores (SDS-PAGE) for Cyro-EM sample preparation (Supplementary Fig. S1). The peak fractions were concentrated to 10–12 mg ml^–1^ for grid preparation.

### Site-Directed mutagenesis and truncations building

Site-directed mutagenesis of the pcDNA3.1(-)-ATP13A2 plasmid was constructed using Mut Express II Fast Mutagenesis Kit (Vazyme Biotech). The resulting pcDNA3.1(-)-ATP13A2-(D513A or K160A; R161A) plasmid DNAs were isolated using the QIAprep Spin Miniprep Kit (Qiagen) and verified by sequence analysis. The vectors expressing ATP13A2 truncations [(1-1155)-strep, (1-1160)-strep, (1-1165)-strep, (1-1170)-strep, (1-1175)-strep] were constructed by adding a stop codon and a Strep-tag sequence to the target sites of pcDNA3.1(-)-ATP13A2 plasmid using Mut Express II Fast Mutagenesis Kit and proofed by sequence analysis.

### Protein purification for biochemical assay

A similar protocol as described above was used for mutant ATP13A2 protein expression and purification. To purified the mutant ATP13A2 (D513A or K160A; R161A), 1-L transfected cells were used for each mutant protein. The lysis buffer, wash buffer, elution buffer and SEC buffer were the same as the buffer using in protein purification for cryo-EM analysis. After SEC purification, the peak fractions of each ATP13A2 mutant proteins were collected and the protein concentration was measured.

### Electron microscopy sample preparation and imaging

The cryo-EM grids were prepared using Vitrobot Mark IV (FEI) at 8 °C and 100% humidity. 4 μL aliquots of samples at a concentration of 10–12 mg ml^–1^ were applied onto glow-discharged holey carbon grids (Quantifoil Au R1.2/1.3, 300 mesh). After a waiting time of 5 s, the grids were blotted for 2–5 s and plunged into liquid ethane for quick freezing. For E1 state ATP13A2 (apo), freshly purified protein samples in SEC buffer were applied immediately. For E2-Pi state ATP13A2, the protein in SEC buffer was incubated with 0.2 mM PA, 0.2 mM PI (3,5)P2, and 2 mM SPM on ice for 1 h before grid preparation and freezing. For E1P-ADP state ATP13A2, the protein in SEC buffer was incubated with 2 mM SPM, 5 mM MgCl_2_, and 5 mM ATP on ice for 30 m before grid preparation. The grids were screened on a Tecnai Arctica microscope (FEI) operated at 200 kV using a Falcon 3 direct electron detector (FEI). The qualified grids were transferred into a Titan Krios microscope (FEI) operated at 300 kV equipped with a Cs image corrector and an energy filter (slit width 20 eV; GIF Quantum LS, Gatan) for data acquisition. Images were recorded using a K3 direct electron detector (Gatan) in a super mode at a nominal magnification of 64, 000 ×, corresponding to a calibrated pixel size of 0.54895 Å. Data acquisition was performed automatically using AutoEMation2.0^52^ in a movie mode, with a frame exposure time of 0.08 s and a total exposure time of 2.56 s, resulting in a total of 32 frames per stack and the total dose rate for each stack was ~ 50 e^–^ Å^–2^. All 32 frames in each stack were aligned and summed using the whole-image motion correction program MotionCor2^53^ and binned to a pixel size of 1.0979. The defocus value of each image, which was set from –1.3 to –1.8 μm during data collection, was determined by Gctf^54^.

### Image processing and 3D reconstruction

To obtain different states of ATP13A2 protein, totally three data sets were collected. For the data set of E1 state, E2-Pi state, and E1P-ADP state ATP13A2, 2425 micrographs (movie stacks), 5976 micrographs, and 2120 micrographs were collected, respectively. For the data set of E1 state ATP13A2 (apo), to generate templates for automatic picking, around 2,000 particles were manually picked and classified by 2D classification in RELION3^55^. After automatic picking and manual micrograph inspection, ~ 1.6 M particles were extracted for subsequent 2D and 3D classification. After three rounds of 2D classification in RELION3, ~ 1.1 M particles from qualified 2D averages were selected for further 3D analysis. Using cryoSPARC^56^ Ab initio reconstruction with no symmetry imposed, an initial model was generated. The 3D classification was then carried out with C1 symmetry using the reference model generated from cryoSPARC, which had been low-pass filters to 60 Å. In total, 350,542 particles with good signals were generated after four rounds of 3D classification, then single-particle Gctf was applied to refine the local defocus parameters of these particles. To get higher resolution, the resultant particles were re-centered for further processing using cryoSPARC. After five round of ab initio reconstruction and the following heterogeneous refinement to further remove bad particles, the final data set of 153,193 particles were used for 3D reconstruction by non-uniform (NU) refinement, and local and global CTF refinement to yield a map at 3.3-Å resolution with C1 symmetry. Similar data processing procedures were applied for data sets of the other two ATP13A2 states. At last, a 3.6-Å resolution map of E2-Pi state ATP13A2 and a 3.0-Å resolution map of E1P-ADP state ATP13A2 were obtained. The local resolution map was calculated using ResMap^57^ and displayed in Chimera^58^. Please refer to Supplementary Figs. S2–S4 for the workflow of image processing.

### Model building

The model of E1P-ADP state ATP13A2 was first built from de novo in COOT^59^ because of its highest resolution. Before model building, models of full-length ATP13A2 were predicted on I-TASSER and Phyre2 online servers^60^. Sequence alignment and secondary structure prediction of ATP13A2 were used to aid the model building. The predicted model of hATP13A2 was docked into the cryo-EM map with a resolution of 3.0 Å in Chimera and manually adjusted in Coot to acquire the atomic model of hATP13A2^58,61^. Model refinement was performed on the main chain of the two atomic models using the real_space_refine module of PHENIX^62^ with secondary structure and geometry restraints to avoid over-fitting. The initial model of the E1 and E2-Pi state of hATP13A2 were generated by fitting separate TMD, A domain, P domain, and N domain of the E1P-ADP ATP13A2 into the maps of E1 and E2-Pi maps by rigid-body fitting. After manual adjustment in COOT, the models were subjected to real-space refinement in PHENIX against the E1 and E2-Pi maps, respectively. Cryo-EM data collection and refinement statistics are shown in Supplementary Table S1.

### ATPase assay

The wild type or mutant ATP13A2 proteins (D513A or K160A; R161A) used for ATPase activity assay were purified as mentioned above. The ATPase activity of ATP13A2 was measured using a commercially available luminescence assay (ADP-Glo™ Max Assay, Promega). Reactions were performed using the reaction buffer from the assay kit in a final volume of 20 μl and at a final condition of 50 mM Tris (PH 8.0), 10 mM MgCl_2_, 1 mM DTT, 0.1% digitonin, various concentrations of the SPM, and 0.018 mg ml^–1^ proteins. Especially, for the lipid groups, 125 μM PA was added in the reaction buffer. Before starting the reaction, proteins and substrates were pre-heated at 37 °C for 5 min. The reactions were carried out for 20 min at 37 °C and were stopped by the addition of the reagent buffer from the assay kit. The next steps were followed by ADP-Glo™ Max Assay Protocols. The 384-well plate (Corning) was then transferred to EnVision system (PerkinElemer) and the luminescence was measured. ATPase rates were determined using linear regression and nonlinear regression of the Michaelis–Menten equation in GraphPad Prism 8.2.1 (GraphPad Software).

### Immunoblotting

As described above, to investigate the function of CTD of ATP13A2, we generated five plasmids by truncating 5, 10, 15, 20, or 25 amino acids starting from the C-terminus of the ATP13A2, respectively. The resulting truncations ATP13A2(1-1155)-strep, ATP13A2(1-1160)-strep, ATP13A2(1-1165)-strep, ATP13A2(1-1170)-strep, and ATP13A2(1-1175)-strep were expressed by transfect 10-ml HEK293F cells for each truncation with a ratio of 10 μg plasmid: 25 μg PEI. After 60 h, transfected cells were collected and lysed in 2 ml lysis buffer containing 25 mM HEPES (pH 7.4), 150 mM NaCl, 2 mM DTT, 1% (w/v) digitonin, and protease inhibitors. The cell lysate was incubated for 2 h at 4 °C with gentle rotation. After centrifugation at 20,000 × g for 15 m, the cell debris was removed and the supernatant was collected. For each protein sample, 10 μL supernatant was mixed with SDS-PAGE loading buffer and incubated at 60 °C for 30 min. The samples were resolved by a 4–20% SDS-PAGE gel (GenScript) and transferred to a PVDF membrane (Millipore). The membrane was first blocked with 5% nonfat dry milk (Bio-Rad) in PBS with 0.1% Tween 20 (PBS-T) for 1 h and then was cut to two slides. The slide containing proteins over 70 kDa was incubated with a mouse anti-Strep monoclonal antibody (Bioeasytech, cat. no. BE2076) at a 1:3,000 dilution for 1 h at room temperature (RT), whereas the other slide containing proteins < 70 kDa was incubated with a mouse anti-β-tubulin monoclonal antibody (Bioeasytech, cat. no. BE0025) at a 1:3,000 dilution for 1 h at RT. The membranes were washed three times with PBS-T for 5 min each. HRP-conjugated goat anti-mouse IgG (Bio-Rad, cat. no. 170-5047) was then added to the membranes at a 1:5,000 dilution for 1 h at RT. The membranes were then washed three more times with PBS-T, and the proteins were detected with enhanced chemiluminescent substrate (Pierce) by Amersham Imager 600 (GE healthcare).

### Lipid overlay assay

Human ATP13A2 isoform A with C-terminus twin Strep-tag in digitonin micelles was purified using the same protocol as mentioned above. The protein was applied on membrane lipid strips and PIP strips (Echelon, P-6001, and P6002) with lipid spots according to the manufacturer’s protocol with minor modifications. In brief, the lipid strips were blocked in 10 ml blocking buffer containing 25 mM HEPES (pH 7.4), 150 mM NaCl, 3% (w/v) BSA and 0.1% (w/v) digitonin at RT for 1 h. The lipid strips were incubated in 10 ml blocking buffer plus 1.5 nM ATP13A2 protein for 1 h at RT with gentle agitation. The protein solution was discarded and the lipid strips were washed with 10 mL wash buffer containing 25 mM HEPES (pH 7.4), 150 mM NaCl and 0.1% (w/v) digitonin three times with gentle agitation for ten minutes each. The lipid strips were then incubated with a mouse anti-Strep antibody (Easybio, cat. no. BE2076) at a 1:3,000 dilution in blocking buffer for 1 h at RT. Three more times wash was applied, then HRP-conjugated goat anti-mouse IgG (Bio-Rad, cat. no. 170-5047) was added to the lipid strips at a 1:5,000 dilution in blocking buffer for 1 h at RT. After the final three-times wash, the protein bound to lipid strips was detected with enhanced chemiluminescent substrate (Pierce) by Amersham Imager 600 (GE healthcare).

## Supplementary information

Supplementary Information

## Data Availability

The 3D cryo-electron microscopy density map and the coordinates of atomic models have been deposited in the Electron Microscopy Data Bank (EMDB) and the Protein Data Bank (PDB) with the following accession codes: EMD-31623 and PDB-7FJM for E1 state; EMD-31626 and PDB-7FJP for E1P-ADP state; and EMD-31627 and PDB-7FJQ for E2-Pi state.
