# Supplementary material for: Cryo-EM structures and transport mechanism of human P5B type ATPase ATP13A2
Source: Cell Discov. 2021 Nov 2;7:106. doi: 10.1038/s41421-021-00334-6 (PMC8564547; doi:10.1038/s41421-021-00334-6)
Supplement: Supplementary file 1 — Supplementary Information [file 41421_2021_334_MOESM1_ESM.pdf]

Supplementary Materials for

Cryo-EM structures and transport mechanism of human P5B type ATPase ATP13A2

Xudong Chen[1*], Mingze Zhou[1*], Sensen Zhang[1*], Jian Yin[1*], Ping Zhang[2], Xujun Xuan[3], Peiyi Wang[4#], Zhiqiang Liu[5#], Boda Zhou[2#], Maojun Yang[1,4,#]

[1]*Ministry of Education Key Laboratory of Protein Science, Tsinghua-Peking Center for Life Sciences, Beijing Advanced Innovation Center for Structural Biology, School of Life Sciences, Tsinghua University, Beijing 100084, China*

[2]*Department of Cardiology, Beijing Tsinghua Changgung Hospital, School of Clinical Medicine, Tsinghua University, Beijing 102218, China.*

[3]*Department of Andrology, The Seventh Affiliated Hospital, Sun Yat-sen University, ShenZhen, 518107, China.*

[4]*Cryo-EM Facility Center, Southern University of Science & Technology, Shenzhen, 518055, China*

[5]*Department of Anesthesiology, Shanghai First Maternity and Infant Hospital, School of Medicine, Tongji University, Shanghai 200092, China*

[*]These authors contribute equally to this work.

[#]To whom correspondence should be addressed.

Peiyi Wang: wangpy@sustech.edu.cn

Zhiqiang Liu: drliuzhiqiang@163.com

Boda Zhou: zhouboda@126.com

Maojun Yang: maojunyang@tsinghua.edu.cn

This file includes

Supplementary Figs. S1 to S10

Supplementary Table S1-S2

Supplementary References

Supplementary figures and Figure legends

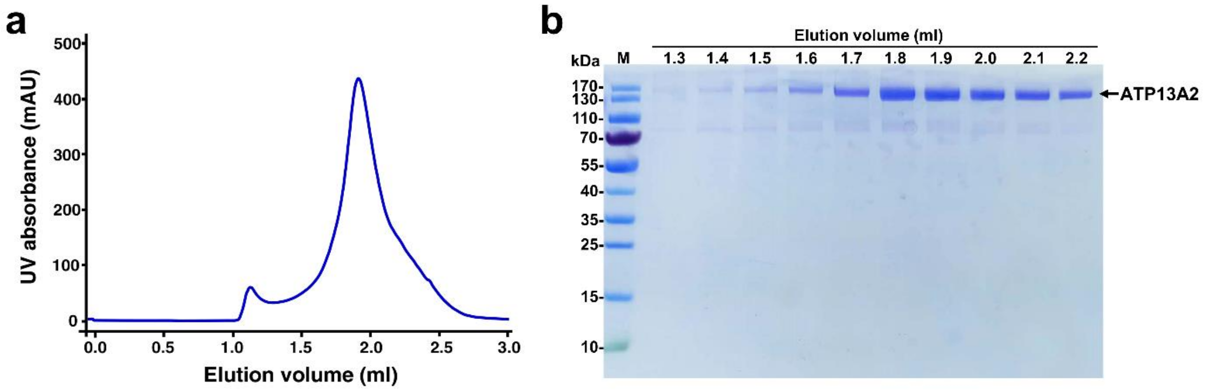

Supplementary Fig. S1 Protein purification of human ATP13A2 for Cryo-EM analysis

a, A representative trace of size-exclusion chromatography of human ATP13A2 by Superose 6 5/150 GL column. UV, ultraviolet.

b, Protein samples of the size-exclusion chromatography fractions were subjected to SDS-PAGE. Fractions of 1.8-2.0 mL (elution volume) were used for cryo-EM sample preparation.

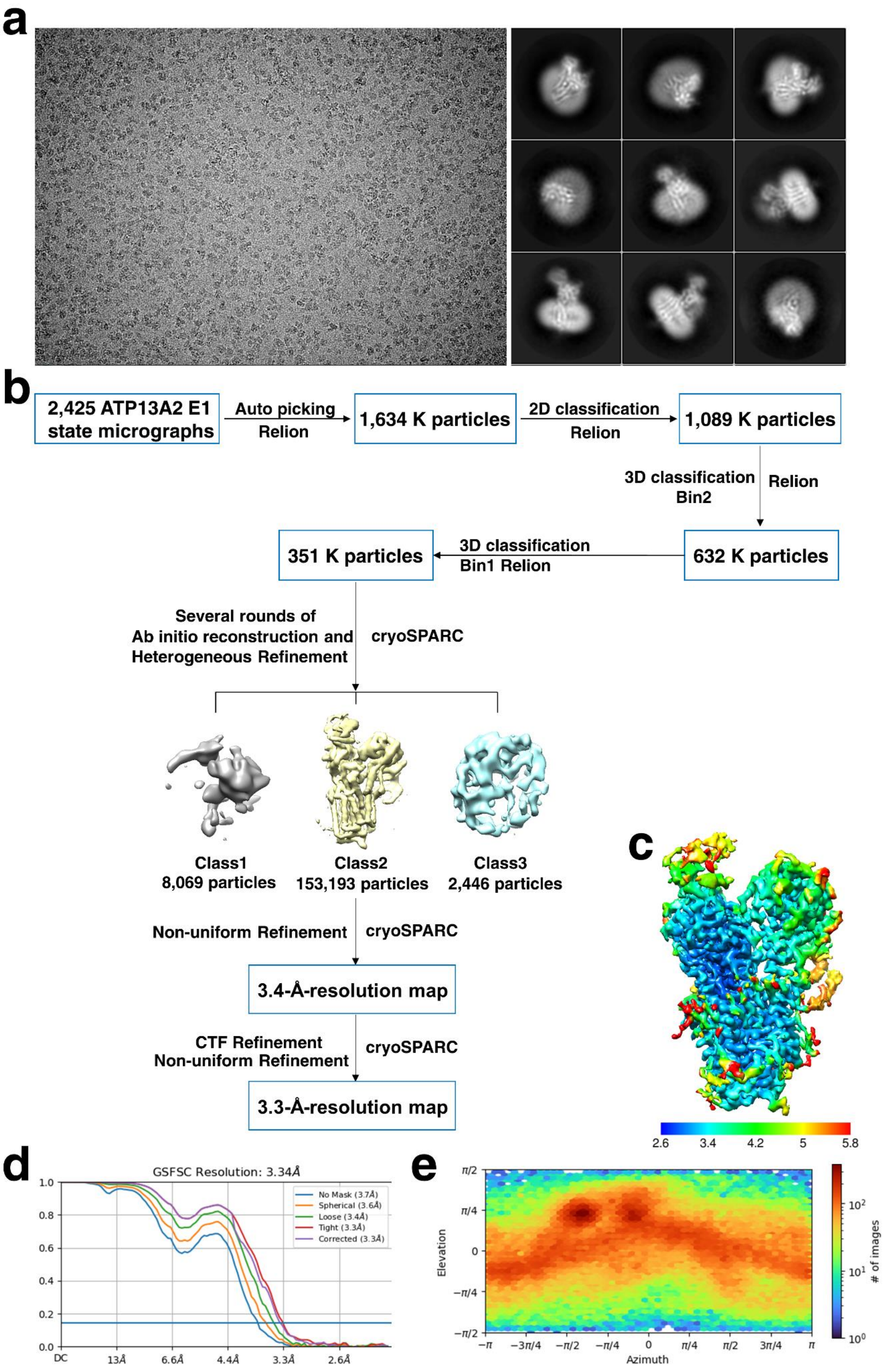

a
b
2,425 ATP13A2 E1 state micrographs
Auto picking Relion
1,634 K particles
2D classification Relion
1,089 K particles
3D classification Bin2
Relion
632 K particles
3D classification Bin1 Relion
351 K particles
Several rounds of Ab initio reconstruction and Heterogeneous Refinement
cryoSPARC
Class1 8,069 particles
Class2 153,193 particles
Class3 2,446 particles
Non-uniform Refinement
cryoSPARC
3.4-Å-resolution map
CTF Refinement Non-uniform Refinement
cryoSPARC
3.3-Å-resolution map
c
2.6
3.4
4.2
5
5.8
d
GSFSC Resolution: 3.34Å
No Mask (3.7Å)
Spherical (3.6Å)
Loose (3.4Å)
Tight (3.3Å)
Corrected (3.3Å)
DC
13Å
6.6Å
4.4Å
3.3Å
2.6Å
e
Elevation
Azimuth
# of images

Supplementary Fig. S2 Reconstruction and structure determination of the E1 state ATP13A2.

a, Representative cryo-EM micrograph and 2D class averages of E1 state ATP13A2.

b, The workflow of single particle analysis for E1 state-ATP13A2 cryo-EM data. In brief, 1,089 k particles were kept after 2D classification, and subjected to five rounds of 3D classification in RELLON3. After further processing using cryoSPARC, a final dataset containing 153 k particles were used for non-uniform refinement and CTF refinement to yield a map at 3.3-Å resolution (see methods for more details).

c, Local resolution map of the final 3D density map.

d, Gold-standard Fourier Shell correlation (FSC) curve of E1 state ATP13A2 after non-uniform refinement. The resolution estimation was based on the criterion of FSC 0.143 cutoff.

e, Distribution of particle orientations in the final 3D reconstruction of the E1 state ATP13A2 structure.

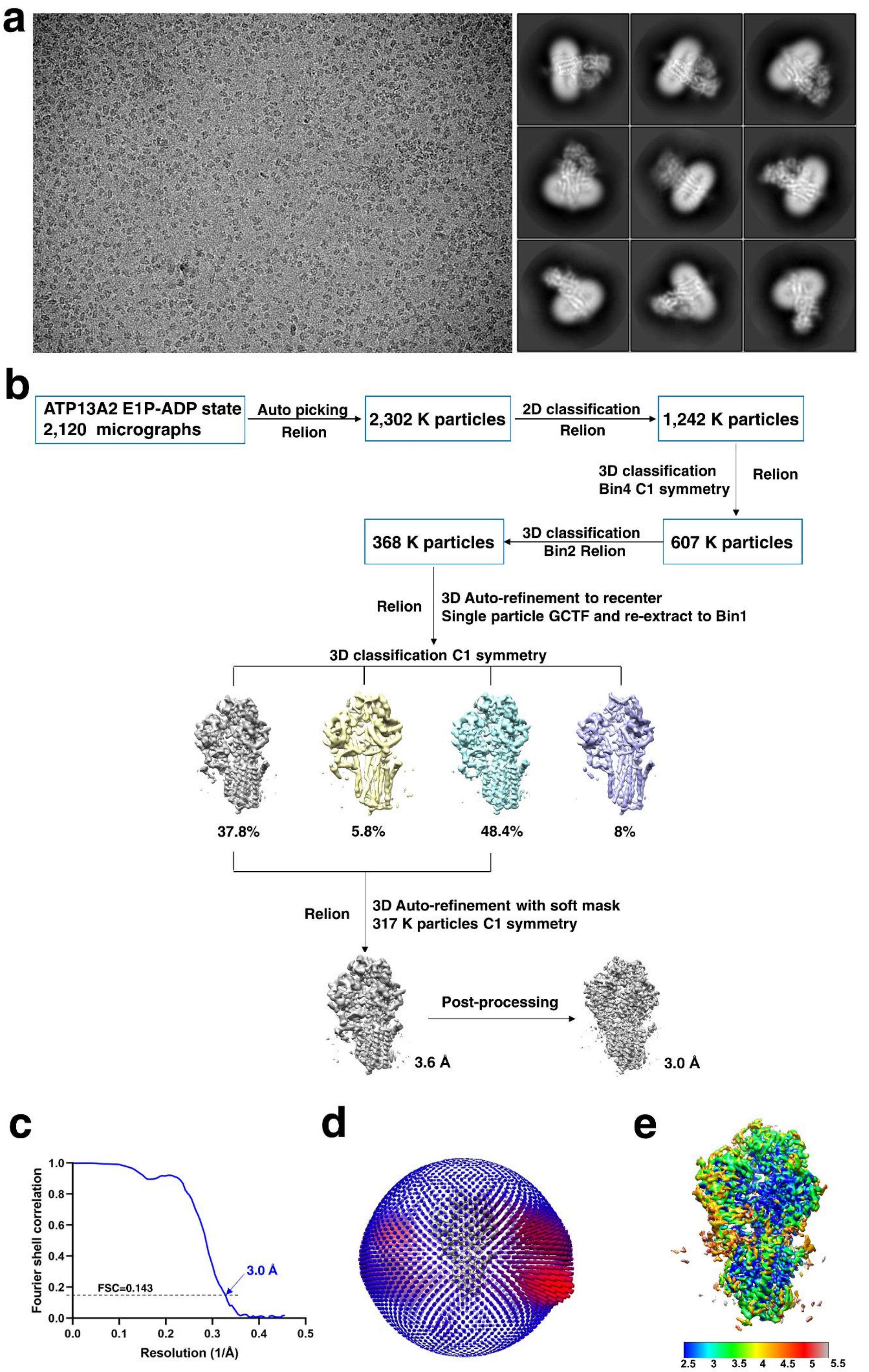
a
b
ATP13A2 E1P-ADP state
2,120 micrographs
Auto picking
Relion
2,302 K particles
2D classification
Relion
1,242 K particles
3D classification
Bin4 C1 symmetry
Relion
607 K particles
3D classification
Bin2 Relion
368 K particles
Relion
3D Auto-refinement to recenter
Single particle GCTF and re-extract to Bin1
3D classification C1 symmetry
37.8%
5.8%
48.4%
8%
Relion
3D Auto-refinement with soft mask
317 K particles C1 symmetry
Post-processing
3.6 Å
3.0 Å
c
d
e
Fourier shell correlation
Resolution (1/Å)
FSC=0.143
3.0 Å
1.0
0.8
0.6
0.4
0.2
0.0
0.0
0.1
0.2
0.3
0.4
0.5
2.5
3
3.5
4
4.5
5
5.5

Supplementary Fig. S3 Reconstruction and structure determination of the E1P-ADP state ATP13A2.

a, Representative cryo-EM micrograph and 2D class averages of E1P-ADP state ATP13A2.

b, The workflow of single-particle analysis for E1P-ADP state ATP13A2 cryo-EM data. In brief, 1,242 k particles were kept after 2D classification, and subjected to five rounds of 3D classification. A final dataset containing 317 k particles were used for high-resolution refinement.

c, Gold-standard Fourier Shell correlation (FSC) curve of E1P-ADP state ATP13A2 after 3D refinement. The resolution estimation was based on the criterion of FSC 0.143 cutoff.

d, Particle orientation distributions in the last iteration of the structural refinement.

e, Local resolution map of the final 3D density map.

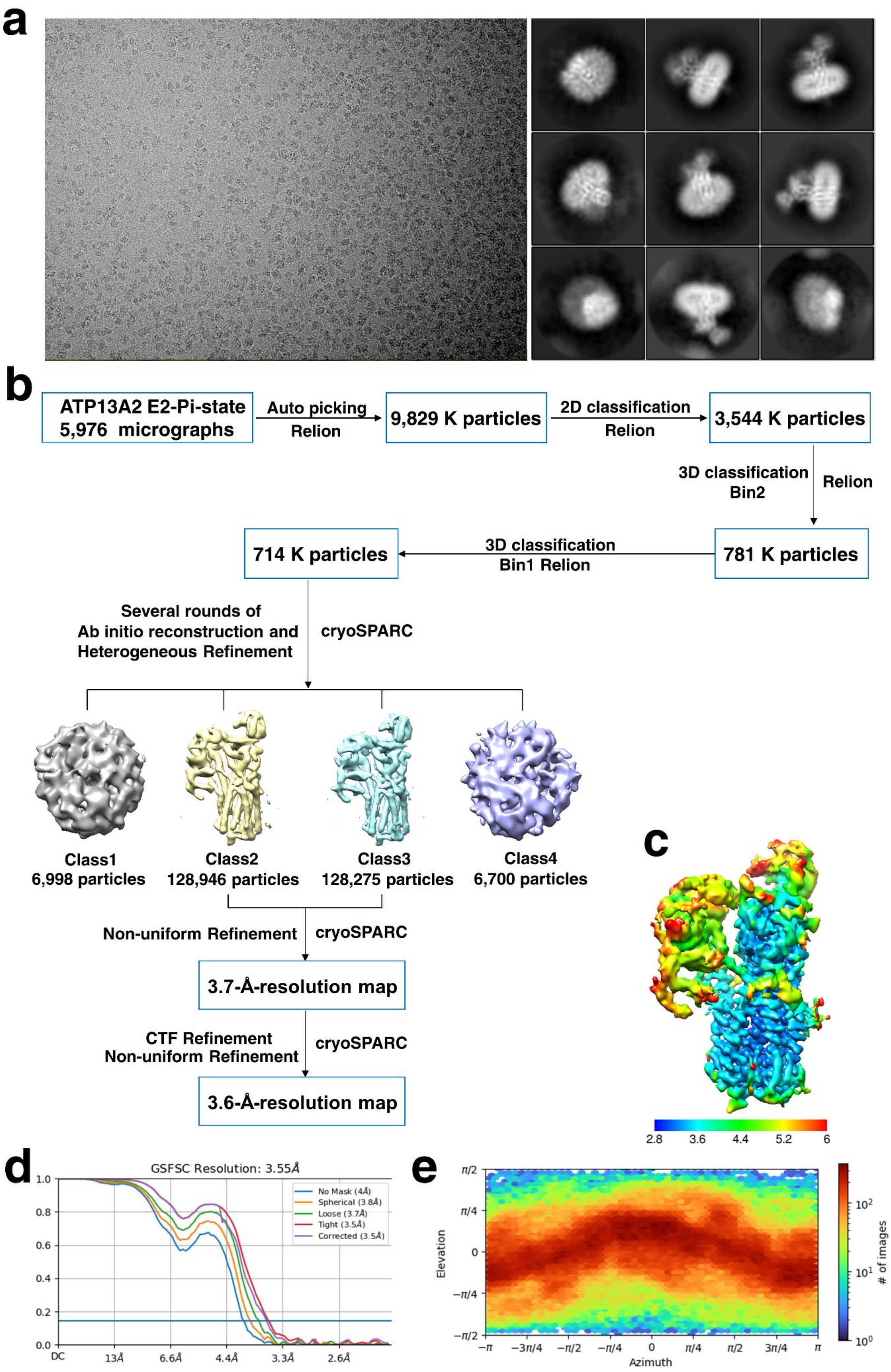

a
b
ATP13A2 E2-Pi-state
5,976 micrographs
Auto picking
Relion
9,829 K particles
2D classification
Relion
3,544 K particles
3D classification
Bin2
Relion
781 K particles
3D classification
Bin1 Relion
714 K particles
Several rounds of
Ab initio reconstruction and
Heterogeneous Refinement
cryoSPARC
Class1
6,998 particles
Class2
128,946 particles
Class3
128,275 particles
Class4
6,700 particles
Non-uniform Refinement
cryoSPARC
3.7-Å-resolution map
CTF Refinement
Non-uniform Refinement
cryoSPARC
3.6-Å-resolution map
c
2.8
3.6
4.4
5.2
6
d
GSFSC Resolution: 3.55Å
No Mask (4Å)
Spherical (3.8Å)
Loose (3.7Å)
Tight (3.5Å)
Corrected (3.5Å)
1.0
0.8
0.6
0.4
0.2
0.0
DC
13Å
6.6Å
4.4Å
3.3Å
2.6Å
e
Elevation
π/2
π/4
0
−π/4
−π/2
−π
−3π/4
−π/2
−π/4
0
π/4
π/2
3π/4
π
Azimuth
# of images
10^2
10^1
10^0

Supplementary Fig. S4 Reconstruction and structure determination of the E2-Pi state ATP13A2.

a, Representative cryo-EM micrograph and 2D class averages of E2-Pi state ATP13A2.

b, The workflow of single particle analysis for E2-Pi state ATP13A2 cryo-EM data. In brief, 9,829 k particles were kept after 2D classification, and subjected to five rounds of 3D classification in RELLON3. After further processing using cryoSPARC, a final dataset containing 257 k particles were used for non-uniform refinement and CTF refinement to yield a map at 3.6-Å resolution.

c, Local resolution map of the final 3D density map.

d, Gold-standard Fourier Shell correlation (FSC) curve of E2-Pi state ATP13A2 after non-uniform refinement. The resolution estimation was based on the criterion of FSC 0.143 cutoff.

e, Distribution of particle orientations in the final 3D reconstruction of the E2-Pi state ATP13A2 structure.

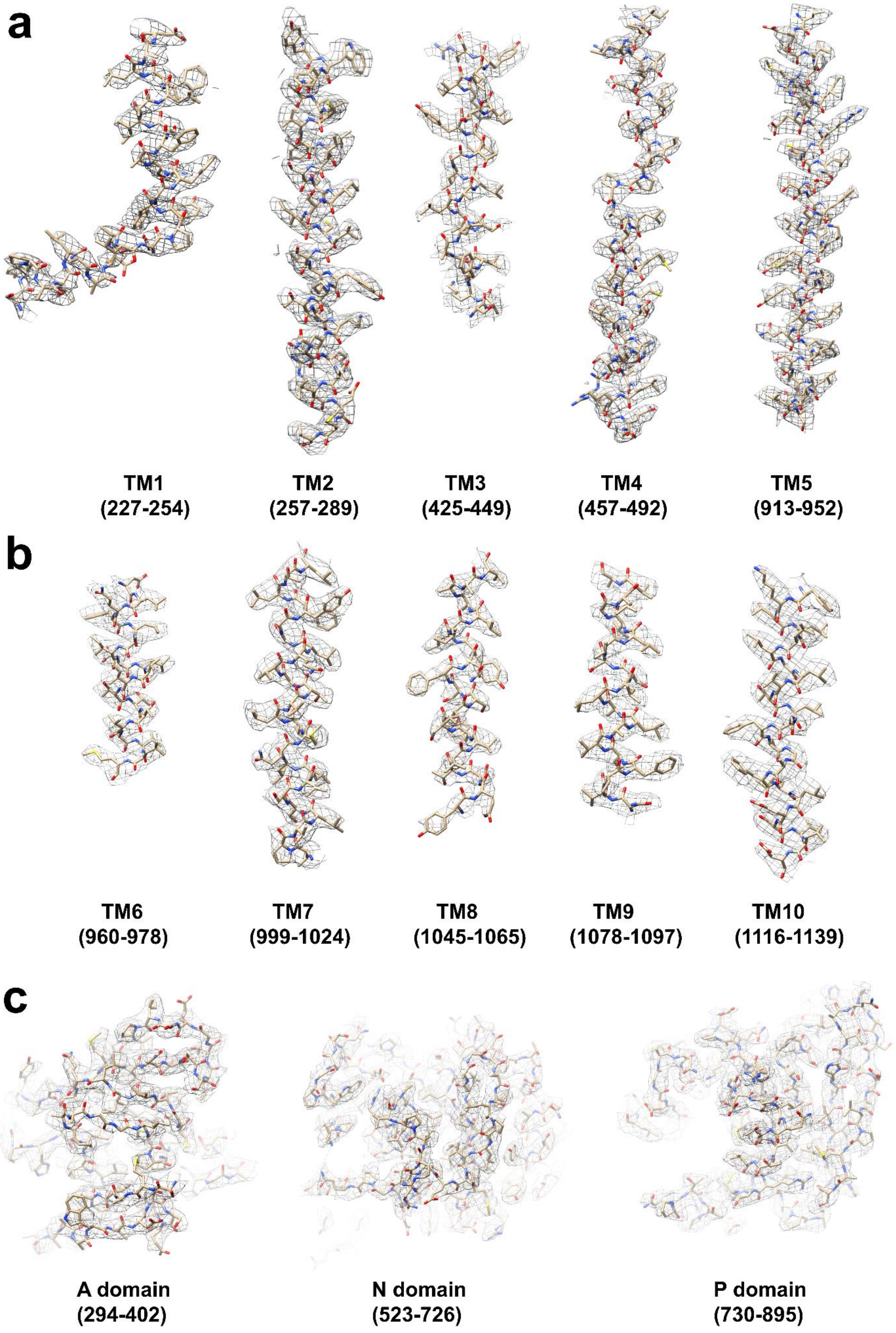
a
TM1
(227-254)
TM2
(257-289)
TM3
(425-449)
TM4
(457-492)
TM5
(913-952)
b
TM6
(960-978)
TM7
(999-1024)
TM8
(1045-1065)
TM9
(1078-1097)
TM10
(1116-1139)
c
A domain
(294-402)
N domain
(523-726)
P domain
(730-895)

Supplementary Fig. S5 Representative cryo-EM densities of ATP13A2.

a,b, Density maps of representative transmembrane regions of ATP13A2. Stick-style atomic models (gold) were fitted into the cryo-EM density maps (gray mesh). The density maps were contoured at 4σ.

c, Density maps of the representative A, N, and P domain region of ATP13A2, similar to the panel **a** and **b**.

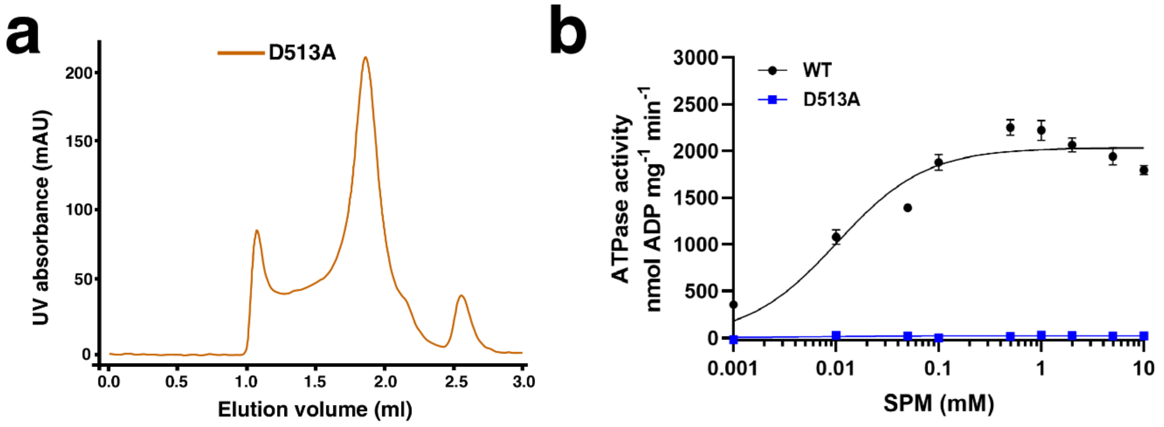

Supplementary Fig. S6 D513 is the catalytic site of ATP13A2.

a, A representative trace of size-exclusion chromatography of ATP13A2 (D513A) by Superose 6 5/150 GL column.

b, ATPase activity of purified ATP13A2 (WT or D513A) under increasing concentrations of SPM. Data are presented as mean ± s.e.m. with n = 4 independent biological experiments. Lines are fitting by nonlinear regression of the Michaelis-Menten equation.

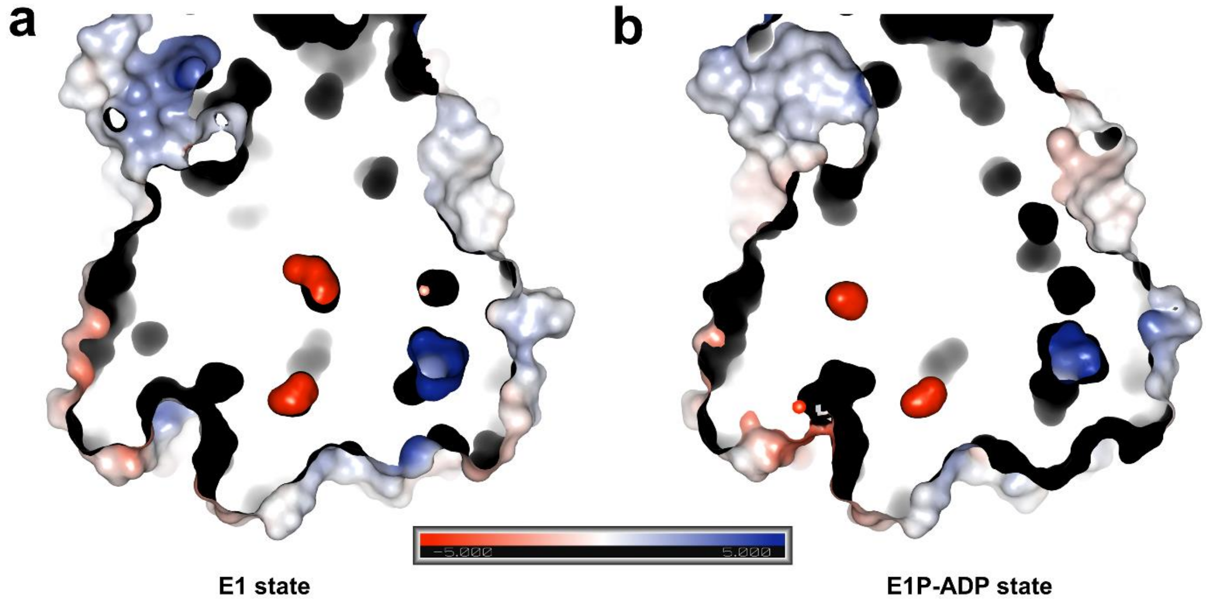

Supplementary Fig. S7 The SPM binding cavity is closed in the E1 state and E1P-ADP state.

a,b Heatmap of the surface electrostatics of TMs shows the closed substrate-binding cavity in E1 state (**a**) and E1P-ADP state (**b**).

a

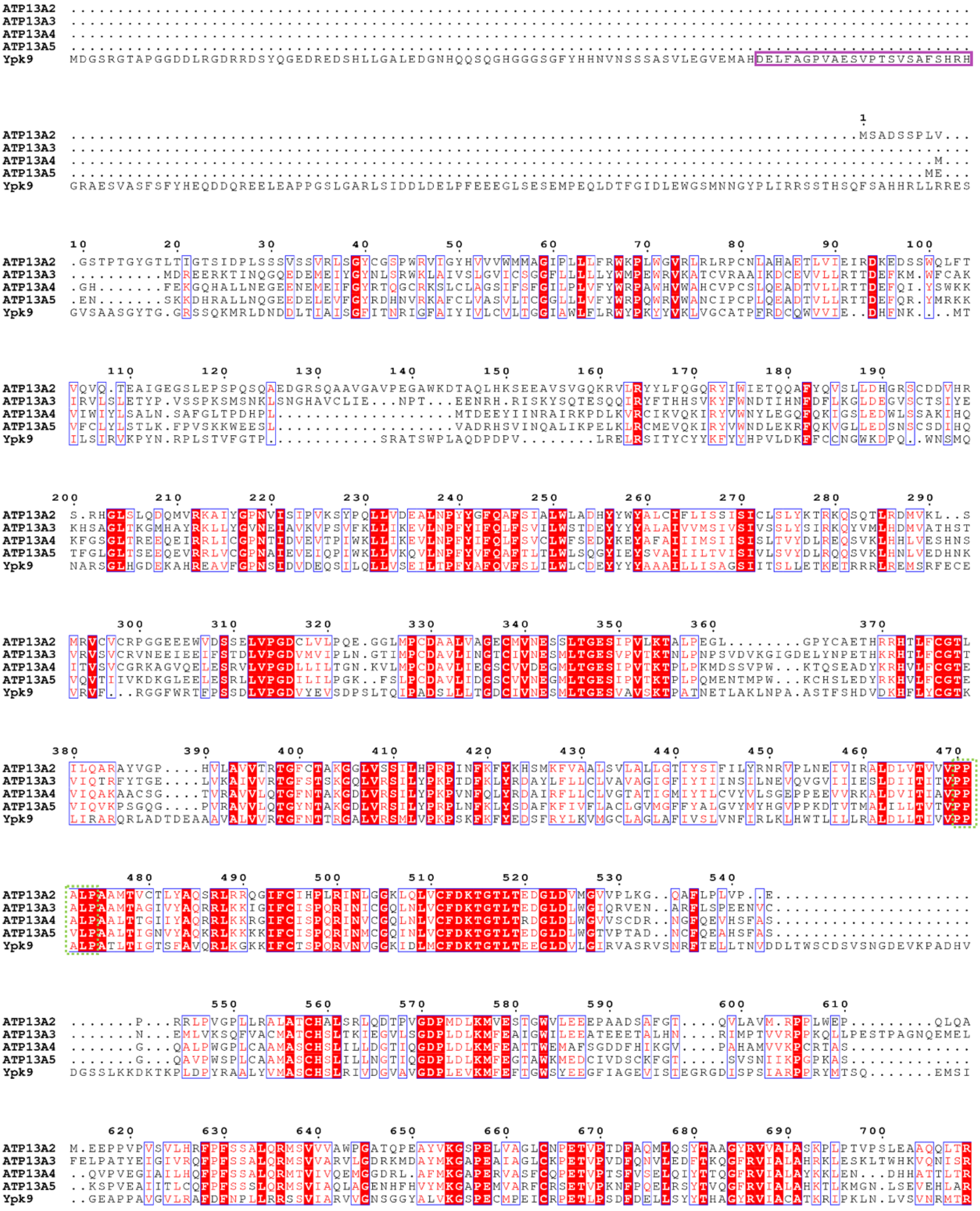

b

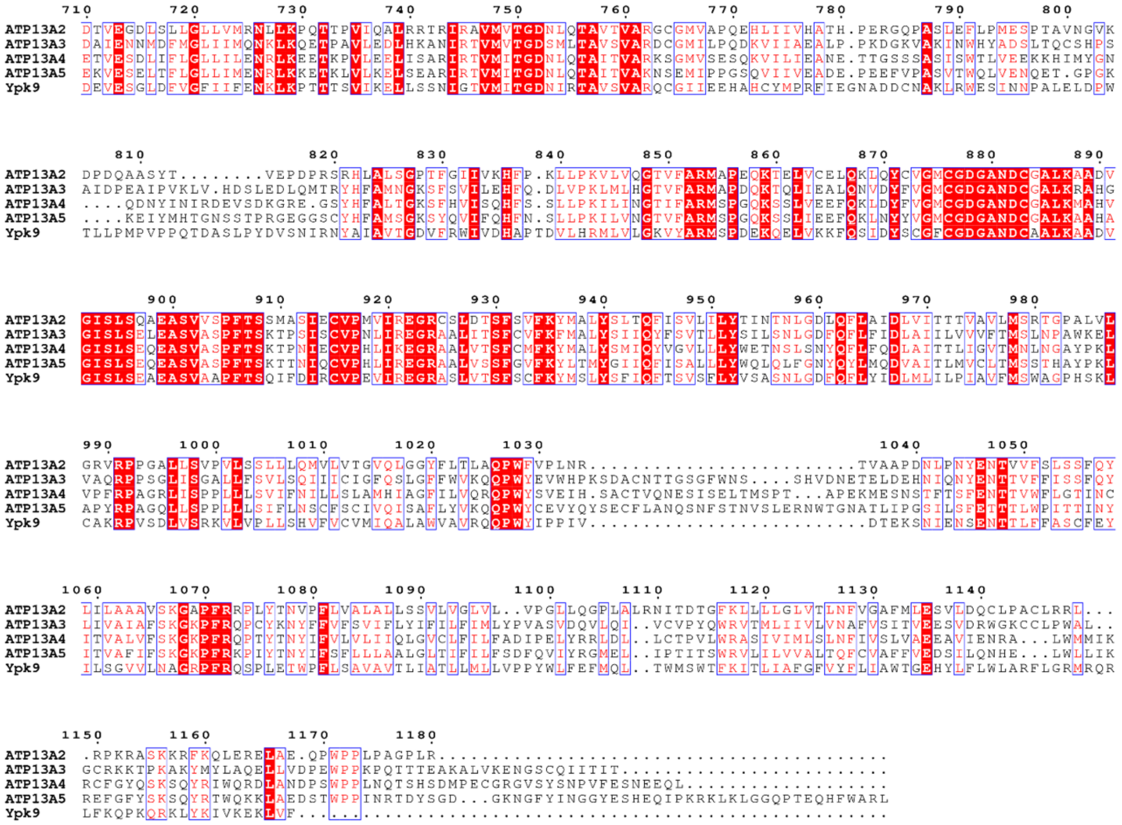

Supplementary Fig. S8 Sequence alignment of human P5B ATPases and yeast Ypk9.

Sequence alignment of human ATP13A2-A5 (Uniprot: Q9NQ11, Q9H7F0, Q4VNC1, and QRVNC0) and yeast Ypk9 (Uniprot: G0S7G9) using ESPript3. Residues are considered as highly similar are colored in red and framed in blue. The sequence in the purple frame indicates the autoinhibitory loop whereas the green dotted box indicates the conserved [PP(A/V)LP] motif.

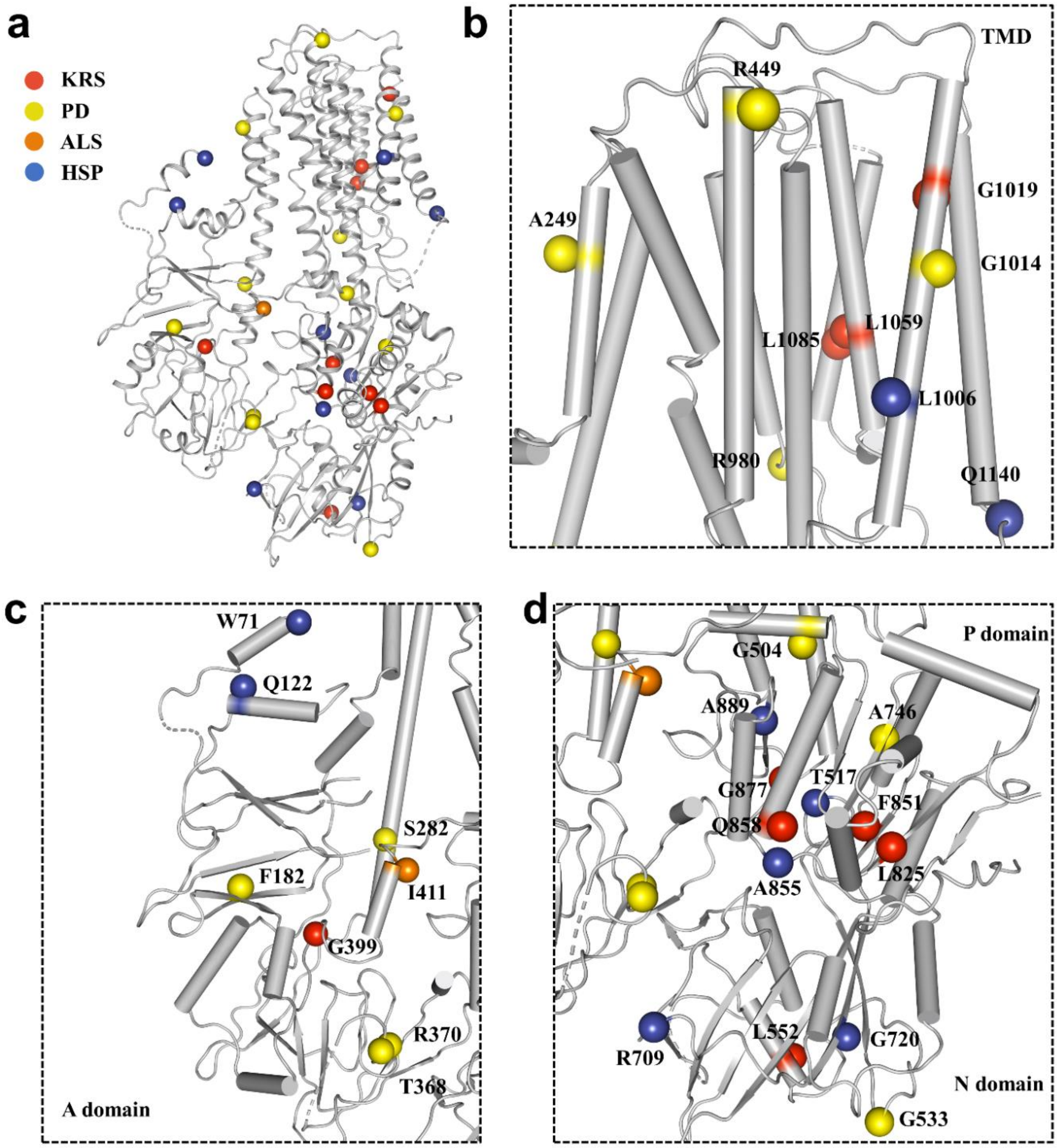

Supplementary Fig. S9 Distribution of ATP13A2 mutations associated with human disease.

a, The affected residues are shown as spheres on the ATP13A2 structure. Kufor-Rakeb syndrome (KRS)-associated mutations are highlighted in red; early-onset Parkinson's disease (PD)-associated mutations in yellow; amyotrophic lateral sclerosis (ALS)-associated mutations in orange; hereditary spastic paraplegia (HSP)-associated mutations in blue. All published mutations are listed in **Supplementary Table S2.**

b, Close-up view of the disease-related mutations on TMD.

c, Close-up view of the disease-related mutations on A domain.

d, Close-up view of the disease-related mutations on N and P domain.

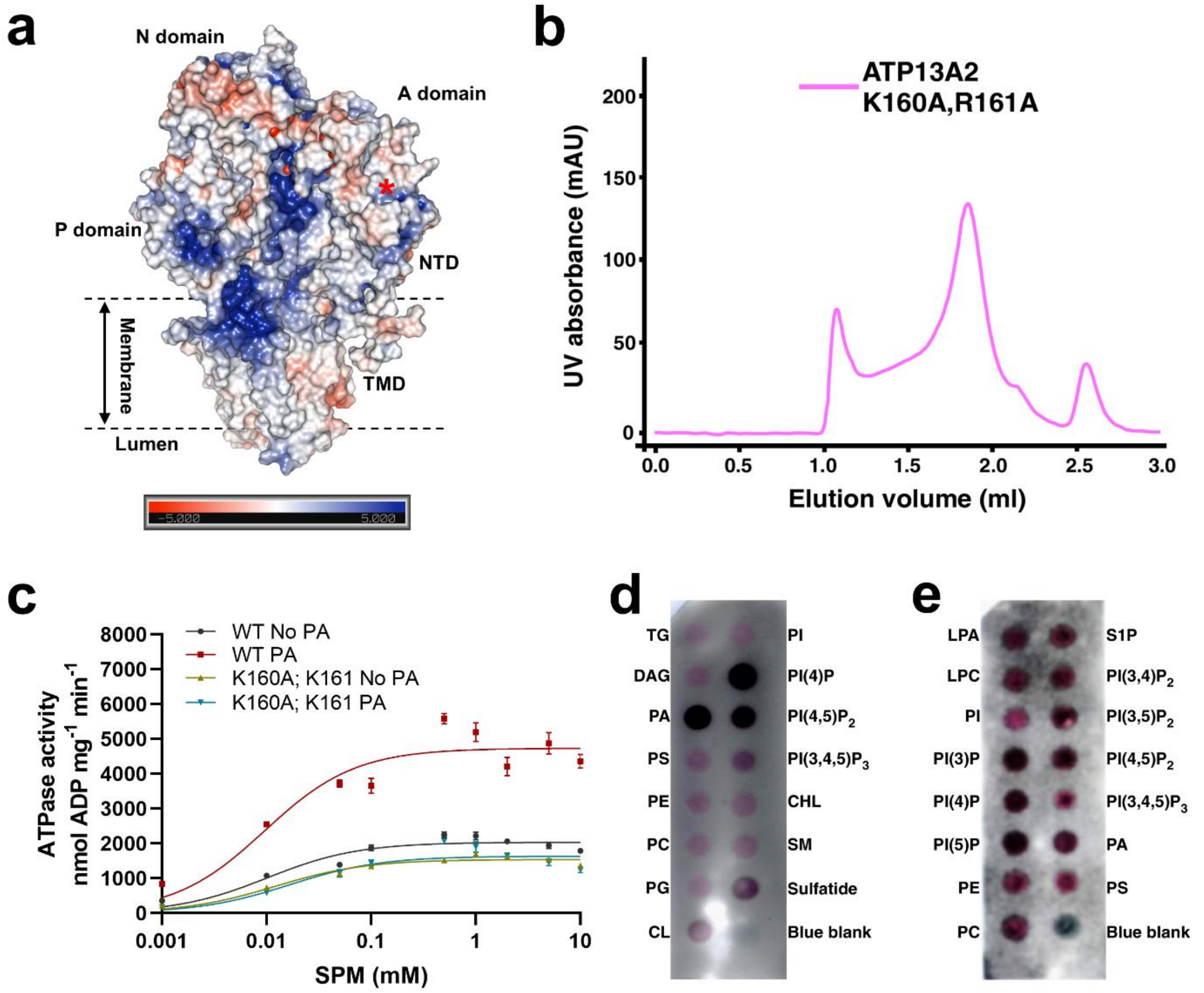

Supplementary Fig. S10 ATP13A2 interacts with regulatory lipids.

a, Heatmap of the surface electrostatics of the E1P-ADP state ATP13A2. The red star indicates the positively charged region in NTD comprising residues K160 and R161, which is likely to be a lipid-binding site.

b, A representative trace of size-exclusion chromatography of ATP13A2 (K160A; R161A) by Superose 6 5/150 GL column.

c, ATPase activity of purified ATP13A2 (WT or K160A/R161A) in digitonin micelles under increasing concentrations of SPM in the presence and absence of PA. Data are presented as mean ± s.e.m. with n = 4 independent biological experiments. lines are fitting by nonlinear regression of the Michaelis-Menten equation.

d,e, Lipid-protein overlay assay with ATP13A2 isoform A on membrane lipid strips (**d**) and PIP strips (**e**) spotted with 22 different lipids. TG, triglyceride; DAG, diacylglycerol; PA,

phosphatidic acid; PS, phosphatidylserine; PE, phosphatidylethanolamine; PC, phosphatidylcholine; PG, phosphatidylglycerol; CL, cardiolipin; PI, phosphatidylinositol; CHL, cholesterol; SM, sphingomyelin; LPA, lysophosphatidic acid; LPC, lysophosphatidylcholine; S1P, sphingosine-1-phosphate;

Supplementary Table S1 | Cryo-EM data collection, refinement and validation statistics

	E1P-ADP	E1	E2-Pi
**Data collection and processing**			
**Magnification**	64,000	64,000	64,000
**Voltage (kV)**	300	300	300
**Electron exposure (e^-/Å^2)**	50	50	50
**Defocus range (μm)**	-1.5 ~ -2.5	-1.5 ~ -2.5	-1.5 ~ -2.5
**Pixel size (Å)**	1.0979	1.0979	1.0979
**Software**	RELION-3.0	RELION-3.0<br>cryoSPARC	RELION-3.0<br>cryoSPARC
**Symmetry imposed**	C1	C1	C1
**Initial particle images (no.)**	2302,382	1634,274	9829,572
**Final particles images (no.)**	317,568	153,193	256,621
**Map resolution (Å)**	3.0	3.3	3.6
**FSC threshold**	0.143	0.143	0.143
**Local map resolution range (Å)**	4.0-2.0		
**Refinement**			
**Software**	PHENIX 1.14	PHENIX1.14	PHENIX 1.14
**Model resolution (Å)**	3.0	3.3	3.6
**FSC threshold**	0.5	0.5	0.5
**Map sharpening *B* factor**	-180	-175	-142
**Model composition**			
**Non-hydrogen atoms**	7248	7095	7109
**Protein residues**	983	973	973
**Ligand**	3	0	1
**Ligand name**	1 ADP, 2 Mg^{2+}		1 SPM
**B factors (Å^2)**			
**Protein**	43.33	114.83	46.42
**Ligand**	62.81	0	13.37
**R.m.s deviations**			
**Bond length (Å)**	0.006	0.006	0.017
**Bond angles (°)**	1.376	1.507	1.900
**Validation**			
**MolProbity score**	1.89	2.01	2.27
**Clashscore**	8.0	5.04	8.43
**Poor rotamers (%)**	0.4	1.93	2.07
**Ramachandran plot**			
**Favored (%)**	92.91	90.79	89.01
**Allowed (%)**	7.09	8.89	10.34
**Disallowed (%)**	0.00	0.32	0.65

Supplementary Table S2 | Pathogenesis mutations of ATP13A2.

cDNA change	Protein change	Disease	Reference	Time
c.3057delC	p.1019GfsX1021	KRS	[1]	2006
c.1306+5G>A	p.G399_L435del	KRS		
c.1632_1653dup22	p.L552QfsX788	KRS		
c.1510G>C	p.G504R	PD	[2]	2007
c.35C>T	p.T12M	PD		
c.1597G>A	p.G533R	PD		
c.546C>A	p.F182L	PD	[3]	2008
c.2236G>A	p.A746T	PD	[4]	2008
c.746C>T	p.A249V	PD	[5]	2009
c.844A>T	p.S282C	PD		
c.2939G>A	p.R980H	PD		
c.1346G>A	p.R449Q	PD		
c.1103_1104insGA	p.T368fsX29	PD	[6]	2010
c.3274A>G	p.G1014S	PD	[7]	2011
c.1108_1120del13	p.R370fsX390	PD	[8]	2011
c.3176T>G	p.L1059R	KRS	[9]	2011
c.3253delC	p.L1085WfsX1088	KRS		
c.2742_2743delTT	p.F851CfsX856	KRS	[10]	2011
c.2629G>A	p.Gly877Arg	KRS	[11]	2011
c.2473C>AA	p.L825fs	KRS	[12]	2012
c.1754G>T	p. A585D	KRS	[13]	2012
c.2429T>G (Isoform B)	p.M810R (Isoform B)	NCL	[14]	2012
c.2762C>T	p.Q858*	KRS	[15]	2014
c.3017_3019del	p.1006_1007del	HSP	[16]	2016
c.2675G>A	p.G892D	HSP	[17]	2016
c.1550C>T	p.T517I	HSP	[18]	2017
c.364C>T	p.Q122*	HSP		
c.1345C>T	p.R449*	HSP		
c.3418C>T	p.Q1140*	HSP		
c.212G>A	p.W71X	HSP	[19]	2018
c.2391delC	p.T798QfsX3	HSP		
c.2629G>A	p.G877R	HSP&KRS	[20]	2019
c.1837G > A,	p.E613*	ALS	[21]	2019
c.1233C > G	p.I411M	ALS		
c.2654C>A	p.A855D	HSP	[22]	2020
c.2126G>C	p.R709T	HSP	[23]	2020
c.2158G>T	p.G720W	HSP		
c.2473_2474insAAdelC	p.L825NfsX32	HSP		

References

1 Ramirez, A. *et al.* Hereditary parkinsonism with dementia is caused by mutations in ATP13A2, encoding a lysosomal type 5 P-type ATPase. *Nat Genet* **38**, 1184-1191, doi:10.1038/ng1884 (2006).

2 Di Fonzo, A. *et al.* ATP13A2 missense mutations in juvenile parkinsonism and young onset Parkinson disease. *Neurology* **68**, 1557-1562, doi:DOI 10.1212/01.wnl.0000260963.08711.08 (2007).

3 Ning, Y. P. *et al.* PARK9-linked parkinsonism in eastern Asia: Mutation detection in ATP13A2 and clinical phenotype. *Neurology* **70**, 1491-1493, doi:10.1212/01.wnl.0000310427.72236.68 (2008).

4 Lin, C. H. *et al.* Novel ATP13A2 variant associated with Parkinson disease in Taiwan and Singapore. *Neurology* **71**, 1727-1732, doi:10.1212/01.wnl.0000335167.72412.68 (2008).

5 Djarmati, A. *et al.* ATP13A2 Variants in Early-Onset Parkinson's Disease Patients and Controls. *Movement Disord* **24**, 2104-2111, doi:10.1002/mds.22728 (2009).

6 Schneider, S. A. *et al.* ATP13A2 Mutations (PARK9) Cause Neurodegeneration with Brain Iron Accumulation. *Movement Disord* **25**, 979-984, doi:10.1002/mds.22947 (2010).

7 Chen, C. M. *et al.* ATP13A2 Variability in Taiwanese Parkinson's Disease. *Am J Med Genet B* **156b**, 720-729, doi:10.1002/ajmg.b.31214 (2011).

8 Fong, C. Y., Rolfs, A., Schwarzbraun, T., Klein, C. & O'Callaghan, F. J. K. Juvenile parkinsonism associated with heterozygous frameshift ATP13A2 gene mutation. *Eur J Paediatr Neuro* **15**, 271-275, doi:10.1016/j.ejpn.2011.01.001 (2011).

9 Park, J. S. *et al.* Pathogenic effects of novel mutations in the P-type ATPase ATP13A2 (PARK9) causing Kufor-Rakeb syndrome, a form of early-onset parkinsonism. *Hum Mutat* **32**, 956-964, doi:10.1002/humu.21527 (2011).

10 Crosiers, D. *et al.* Juvenile dystonia-parkinsonism and dementia caused by a novel ATP13A2 frameshift mutation. *Parkinsonism Relat D* **17**, 135-138, doi:10.1016/j.parkreldis.2010.10.011 (2011).

11 Santoro, L. *et al.* Novel ATP13A2 (PARK9) homozygous mutation in a family with marked phenotype variability. *Neurogenetics* **12**, 33-39, doi:10.1007/s10048-010-0259-0 (2011).

12 Eiberg, H. *et al.* Novel mutation in ATP13A2 widens the spectrum of Kufor-Rakeb syndrome (PARK9). *Clin Genet* **82**, 256-263, doi:10.1111/j.1399-0004.2011.01745.x (2012).

13 Zhu, L. H. *et al.* Lack of association between three single nucleotide polymorphisms in the PARK9, PARK15, and BST1 genes and Parkinson's disease in the northern Han Chinese population. *Chinese Med J-Peking* **125**, 588-592, doi:10.3760/cma.j.issn.0366-6999.2012.04.006 (2012).

14 Bras, J., Verloes, A., Schneider, S. A., Mole, S. E. & Guerreiro, R. J. Mutation of the parkinsonism gene ATP13A2 causes neuronal ceroid-lipofuscinosis. *Hum Mol Genet* **21**, 2646-2650, doi:10.1093/hmg/dds089 (2012).

15 Malakouti-Nejad, M. *et al.* Identification of p.Gln858*in ATP13A2 in two EOPD patients and presentation of their clinical features. *Neurosci Lett* **577**, 106-111, doi:10.1016/j.neulet.2014.06.023 (2014).

16 Kara, E. *et al.* Genetic and phenotypic characterization of complex hereditary spastic paraplegia. *Brain* **139**, 1904-1918, doi:10.1093/brain/aww111 (2016).

17 van de Warrenburg, B. P. *et al.* Clinical exome sequencing for cerebellar ataxia and spastic paraplegia uncovers novel gene-disease associations and unanticipated rare disorders. *Eur J Hum Genet* **24**, 1460-1466, doi:10.1038/ejhg.2016.42 (2016).

18 Estrada-Cuzcano, A. *et al.* Loss-of-function mutations in the ATP13A2/PARK9 gene cause complicated hereditary spastic paraplegia (SPG78). *Brain* **140**, 287-305, doi:10.1093/brain/aww307 (2017).

19 Jamil, M., Siddiqui, A. W., Sollinger, A. & Ferrara, J. ATP13A2-Related Heriditary Spastic Paraplegia

(HSP). *Neurology* **90** (2018).

20 Erro, R., Picillo, M., Manara, R., Pellecchia, M. T. & Barone, P. From PARK9 to SPG78: The clinical spectrum of ATP13A2 mutations. *Parkinsonism Relat D* **65**, 272-273, doi:10.1016/j.parkreldis.2019.05.025 (2019).

21 Spataro, R. *et al.* Mutations in ATP13A2 (PARK9) are associated with an amyotrophic lateral sclerosis-like phenotype, implicating this locus in further phenotypic expansion. *Hum Genomics* **13**, 19, doi:10.1186/s40246-019-0203-9 (2019).

22 Odake, Y. *et al.* Identification of a novel mutation in ATP13A2 associated with a complicated form of hereditary spastic paraplegia. *Neurol-Genet* **6**, doi:ARTN e51410.1212/NXG.0000000000000514 (2020).

23 Estiar, M. A. *et al.* Clinical and genetic analysis of ATP13A2 in hereditary spastic paraplegia expands the phenotype. *Mol Genet Genom Med* **8**, doi:ARTN e105210.1002/mgg3.1052 (2020).
